# Cardiac Rehabilitation in German Speaking Countries of Europe—Evidence-Based Guidelines from Germany, Austria and Switzerland LLKardReha-DACH—Part 2

**DOI:** 10.3390/jcm10143071

**Published:** 2021-07-12

**Authors:** Bernhard Schwaab, Birna Bjarnason-Wehrens, Karin Meng, Christian Albus, Annett Salzwedel, Jean-Paul Schmid, Werner Benzer, Matthes Metz, Katrin Jensen, Bernhard Rauch, Gerd Bönner, Patrick Brzoska, Heike Buhr-Schinner, Albrecht Charrier, Carsten Cordes, Gesine Dörr, Sarah Eichler, Anne-Kathrin Exner, Bernd Fromm, Stephan Gielen, Johannes Glatz, Helmut Gohlke, Maurizio Grilli, Detlef Gysan, Ursula Härtel, Harry Hahmann, Christoph Herrmann-Lingen, Gabriele Karger, Marthin Karoff, Ulrich Kiwus, Ernst Knoglinger, Christian-Wolfgang Krusch, Eike Langheim, Johannes Mann, Regina Max, Maria-Inti Metzendorf, Roland Nebel, Josef Niebauer, Hans-Georg Predel, Axel Preßler, Oliver Razum, Nils Reiss, Daniel Saure, Clemens von Schacky, Morten Schütt, Konrad Schultz, Eva-Maria Skoda, Diethard Steube, Marco Streibelt, Martin Stüttgen, Michaela Stüttgen, Martin Teufel, Hansueli Tschanz, Heinz Völler, Heiner Vogel, Ronja Westphal

**Affiliations:** 1 Curschmann Klinik, D-23669 Timmendorfer Strand, Germany; 2 Medizinische Fakultät, Universität zu Lübeck, D-23562 Lübeck, Germany; 3 Institute for Cardiology and Sports Medicine, Department of Preventive and Rehabilitative Sport- and Exercise Medicine, German Sportuniversity Cologne, D-50933 Köln, Germany; Bjarnason@dshs-koeln.de (B.B.-W.); Predel@dshs-koeln.de (H.-G.P.); 4 Institute for Clinical Epidemiology and Biometry (ICE-B), University of Würzburg, D-97080 Würzburg, Germany; k.meng@uni-wuerzburg.de; 5 Department of Psychosomatics and Psychotherapy, Faculty of Medicine, University Hospital, D-50937 Köln, Germany; Christian.albus@uk-koeln.de; 6 Department of Rehabilitation Medicine, Faculty of Health Sciences Brandenburg, University of Potsdam, D-14469 Potsdam, Germany; annett.salzwedel@fgw-brandenburg.de (A.S.); saraheichler@t-online.de (S.E.); or Heinz.Voeller@klinikamsee.com (H.V.); 7 Klinik Barmelweid AG, CH-5017 Barmelweid, Switzerland; Jean-Paul.Schmid@barmelweid.ch; 8 Reha-Klinik Montafon, A-6780 Schruns, Austria; wbenzer@cable.vol.at; 9 Institute of Medical Biometry and Informatics (IMBI), University of Heidelberg, D-69120 Heidelberg, Germany; matthes.metz@gmail.com (M.M.); Meinhard.kieser@imbi.uni-heidelberg.de (K.J.); dsaure55@hotmail.de (D.S.); 10 Institut für Herzinfarktforschung Ludwigshafen, IHF, D-67063 Ludwigshafen am Rhein, Germany; Rauch.B@t-online.de; 11 Zentrum für ambulante Rehabilitation, ZAR Trier GmbH, D-54292 Trier, Germany; 12 Medizinische Fakultät, Albert-Ludwigs-Universität zu Freiburg, D-79104 Freiburg, Germany; boenner.mail@t-online.de; 13 Fakultät für Gesundheit, Universität Witten/Herdecke, Lehrstuhl für Versorgungsforschung, D-58448 Witten, Germany; patrick.brzoska@uni-wh.de; 14 Ostseeklinik Schönberg-Holm, D-24217 Schönberg-Holm, Germany; hbuhr-schinner@t-online.de; 15 Private Practice, D-01328 Dresden, Germany; albrecht.charrier@t-online.de; 16 Gollwitzer-Meier-Klinik, D-32545 Bad Oeynhausen, Germany; sy.ca.cordes@gmail.com; 17 Alexianer St. Josefs-Krankenhaus Potsdam, D-14472 Potsdam, Germany; G.Doerr@alexianer.de; 18 Klinikum Lippe GmbH, Standort Detmold, D-32756 Detmold, Germany; Anne-Kathrin.Exner@klinikum-lippe.de (A.-K.E.); Stephan.Gielen@klinikum-lippe.de (S.G.); 19 REHA-Klinik Sigmund Weil, D-76669 Bad Schönborn, Germany; b.fromm@sigmund-weil-klinik.de; 20 Reha-Zentrum Seehof der Deutschen Rentenversicherung Bund, D-14513 Teltow, Germany; jglatz@t-online.de (J.G.); Dr.med.Eike.Langheim@drv-bund.de (E.L.); 21 Private Practice, D-79282 Ballrechten-Dottingen, Germany; h.gohlke@t-online.de; 22 Library Department, University Medical Centre Mannheim, D-68167 Mannheim, Germany; grillimaurizio@posteo.de; 23 Department für Humanmedizin, Private Universität Witten/Herdecke GmbH, D-58455 Witten, Germany; d.gysan@gesundesherz.de; 24 LMU München, Institut für Medizinische Psychologie, D-80336 München, Germany; ursula.haertel@med.uni-muenchen.de; 25 Private Practice, D-88316 Isny im Allgäu, Germany; harry.hahmann@uni-ulm.de; 26 Department of Psychosomatic Medicine and Psychotherapy, University of Göttingen Medical Center and German Center for Cardiovascular Research (DZHK), Partner Site Göttingen, D-37075 Göttingen, Germany; cherrma@gwdg.de; 27 Private Practice, D-69121 Heidelberg, Germany; kargerga@freenet.de; 28 Private Practice, D-58332 Schwelm, Germany; karoffm@t-online.de; 29 Private Practice, D-14169 Berlin, Germany; dr.ulrich.kiwus@t-online.de; 30 Kirchberg-Klinik, D-37431 Bad Lauterberg, Germany; knoglinger@kirchbergklinik.de; 31 Private Practice, D-67059 Ludwigshafen, Germany; Christian-Wolfgang.Krusch@arbeitsagentur.de; 32 KfH Nierenzentrum, D-80804 München, Germany; prof.j.mann@gmail.com; 33 Zentrum für Rheumatologie, Drs. Dornacher/Schmitt/Max/Lutz, D-69115 Heidelberg, Germany; DrReginaMax@mail.de; 34 Cochrane Metabolic and Endocrine Disorders Group, Institute of General Practice, Medical Faculty of the Heinrich-Heine University, D-40225 Düsseldorf, Germany; maria-inti.metzendorf@med.uni-duesseldorf.de; 35 Hermann-Albrecht-Klinik METTNAU, Reha-Einrichtungen der Stadt Radolfzell, D-7385 Radolfzell, Germany; roland.nebel@mettnau.com; 36 Universitätsinstitut für Präventive und Rehabilitative Sportmedizin, Uniklinikum Salzburg, Paracelsus Medizinische Privatuniversität, A-5020 Salzburg, Austria; j.niebauer@salk.at; 37 Privatpraxis für Kardiologie, Sportmedizin, Prävention, Rehabilitation, D-81675 München, Germany; axel.pressler@kardiologie-mit-herz.de; 38 Epidemiologie und International Public Health, Fakultät für Gesundheitswissenschaften, Universität Bielefeld, D-33615 Bielefeld, Germany; oliver.razum@uni-bielefeld.de; 39 Schüchtermann-Schiller’sche Kliniken, D-49214 Bad Rothenfelde, Germany; NReiss@schuechtermann-klinik.de; 40 Omegametrix, D-82152 Martinsried, Germany; c.vonschacky@omegametrix.eu; 41 Diabetologische Schwerpunktpraxis, D-23552 Lübeck, Germany; morten.schuett@diabetes-luebeck.de; 42 Klinik Bad Reichenhall, Zentrum für Rehabilitation, Pneumologie und Orthopädie, D-83435 Bad Reichenhall, Germany; konradschultz@t-online.de; 43 Clinic for Psychosomatic Medicine and Psychotherapy, LVR University Hospital, University of Duisburg-Essen, D-45147 Essen, Germany; eva-maria.skoda@uni-due.de (E.-M.S.); martin.teufel@uni-due.de (M.T.); 44 Private Practice, D-14055 Berlin, Germany; professor.steube@gmx.de; 45 Department for Rehabilitation Research, German Federal Pension Insurance, D-10704 Berlin, Germany; marco.streibelt@drv-bund.de; 46 Praxis Martin Stüttgen, D-67157 Wachenheim, Germany; Martin@Praxis-Stuettgen.de; 47 ZAR Ludwigshafen Klinikum, D-67063 Ludwigshafen am Rhein, Germany; Michaela.stuettgen@zar-ludwigshafen-klinikum.de; 48 Berner Reha Zentrum, CH-3625 Heiligenschwendi, Switzerland; h.tschanz@rehabern.ch; 49 Klinik am See, D-15562 Rüdersdorf, Germany; 50 Abteilung für Medizinische Psychologie und Psychotherapie, Medizinische Soziologie und Rehabilitationswissenschaften, Universität Würzburg, D-97070 Würzburg, Germany; h.vogel@uni-wuerzburg.de; 51 Herzzentrum Segeberger Kliniken, D-23795 Bad Segeberg, Germany; ronja.westphal@segebergerkliniken.de

**Keywords:** cardiac rehabilitation, scientific guidelines, secondary prevention, physical activity, exercise training, psychological interventions, education, gender, frailty, migration, old patients, young patients, tele-medicine, home-based-rehabilitation

## Abstract

Background: Scientific guidelines have been developed to update and harmonize exercise based cardiac rehabilitation (ebCR) in German speaking countries. Key recommendations for ebCR indications have recently been published in part 1 of this journal. The present part 2 updates the evidence with respect to contents and delivery of ebCR in clinical practice, focusing on exercise training (ET), psychological interventions (PI), patient education (PE). In addition, special patients’ groups and new developments, such as telemedical (Tele) or home-based ebCR, are discussed as well. Methods: Generation of evidence and search of literature have been described in part 1. Results: Well documented evidence confirms the prognostic significance of ET in patients with coronary artery disease. Positive clinical effects of ET are described in patients with congestive heart failure, heart valve surgery or intervention, adults with congenital heart disease, and peripheral arterial disease. Specific recommendations for risk stratification and adequate exercise prescription for continuous-, interval-, and strength training are given in detail. PI when added to ebCR did not show significant positive effects in general. There was a positive trend towards reduction in depressive symptoms for “distress management” and “lifestyle changes”. PE is able to increase patients’ knowledge and motivation, as well as behavior changes, regarding physical activity, dietary habits, and smoking cessation. The evidence for distinct ebCR programs in special patients’ groups is less clear. Studies on Tele-CR predominantly included low-risk patients. Hence, it is questionable, whether clinical results derived from studies in conventional ebCR may be transferred to Tele-CR. Conclusions: ET is the cornerstone of ebCR. Additional PI should be included, adjusted to the needs of the individual patient. PE is able to promote patients self-management, empowerment, and motivation. Diversity-sensitive structures should be established to interact with the needs of special patient groups and gender issues. Tele-CR should be further investigated as a valuable tool to implement ebCR more widely and effectively.

## 1. Introduction

Exercise based cardiac rehabilitation (ebCR) is a well-accepted treatment for secondary prevention of cardiovascular diseases. Implementation and delivery of ebCR, however, still considerably differ between countries. Therefore, evidence-based guidelines for ebCR in clinical practice of Germany, Austria and Switzerland were updated and harmonized. The condensed version of these guidelines (LLKardReha-D-A-CH) in English language are presented in two parts. Part 1 [1], describes the methodology of guideline development and the evidence of ebCR for a broad spectrum of clinical indications. Part 2, as presented in this review, concentrates on most important contents of ebCR, such as exercise training, psychological interventions and patient education, with their application in clinical practice. In addition, particular needs of special patients and the potential of upcoming forms of ebCR, such as tele-rehabilitation and home-based CR, are discussed in Part 2 as well.

## 2. Methods

Initiation, organization, and methodology of evidence generation, applied in the Cardiac Rehabilitation Guidelines of German speaking countries in Europe (Germany, Austria, Switzerland; D-A-CH), have been described in detail in Part 1 [1]. The “Grades of recommendation” (see Table 1) were based on the scientific evidence as derived from the literature and a formal consensus process of all members of the steering committee in accordance with the “GRADE Evidence-to-Decision framework” and supervised by the AWMF (“Association of the Scientific Medical Societies in Germany”). The “degree of consensus” is expressed in percentages of all steering committee members participating in the consensus generating process. The “classification of scientific evidence” followed the definitions of SIGN (Scottish Intercollegiate Guidelines Network) and was only applicated in chapters classified by “S3” (for details, see Part 1; [1]).

## 3. Results and Evidence-Based Recommendations for Contents and Application of ebCR

In the following, the recommendations for most important contents of ebCR and their application in clinical practice, as well as particular needs of special patients groups, including gender issues, and the potential of new forms of ebCR like tele-rehabilitation and home-based ebCR, are discussed. The contents being evaluated can be categorized as follows:

- Physical activity and exercise training in different patient cohorts;
- Psychological interventions;
- Patient education;
- Special patient groups;
- Telemedical- and home-based-rehabilitation;

### 3.1. Physical Activity and Exercise Training in General

The following recommendations are based on most recent and topic-related scientific guidelines and by a semi-structured evaluation of scientific literature (classification: S2k; see Methods in Part 1; [1]).

Physical activity (PA) and exercise training (ET) are the corner stones of contemporary cardiac rehabilitation programs resulting in the worldwide accepted term of exercise based cardiac rehabilitation (ebCR). Indications for ET in patients with cardiovascular diseases have been described in Part 1 [1]. The following chapter integrates the practical aspects of PA and ET in this patient cohort.

#### 3.1.1. Recommendations

- All cardiovascular patients in a stable clinical stage are recommended to participate in supervised, individually adapted exercise training as part of ebCR [2,3,4,5,6,7,8,9]. (↑↑ 100%)
- It is recommended (↑↑ 100%)
  - to perform a detailed risk-evaluation including stress test before the start of any exercise measures
  - to provide all patients with targeted individualized motivation and instruction to promote physical activity in everyday life and independent, individually adapted exercise training during leisure time [2,3,4,5,6,7,8,9].
  - to motivate all cardiovascular patients to participate in an exercise based long-term maintenance program [4,5,6,7,8].

#### 3.1.2. Scientific Evidence

Well documented evidence confirms the prognostic significance of ebCR, especially in patients after acute coronary syndrome (ACS) and coronary artery bypass grafting (CABG) [10,11,12]. Favourable effects on prognosis can only be expected with sufficient training volume:

- total exercise volume to be ≥1.000 min as calculated by the “number of weeks” times “exercise sessions per week” times “exercise duration per session in minutes”,
- the number of rehabilitation sessions including exercise, information, education and psychosocial interventions to be ≥36.

The overall incidence of severe exercise-induced cardiovascular complications during supervised monitored exercise training within ebCR is very low [2,13,14]. For risk stratification, see Table 2, and for contraindications, see Data S1 in Supplements [15].

Aerobic endurance training is a core component of [2,4,5,7,8,16]. Its multiple beneficial effects on the symptomatology and progression of various cardiac diseases i.e., CAD [17,18,19], HFrEF [20,21,22,23,24], and after heart transplantation [25,26,27] are well documented. Within ebCR, the moderate continuous aerobic endurance training (MCT) is standard [2,4,5,6,8,16]. (see Table 3) The use of interval training (IT), with very short intervals [20–30 s), in patients with significantly reduced exercise capacity [28] is well established in ebCR.

A recent meta-analysis showed a significant association between hand strength, knee extensor strength, and all-cause mortality. Numerous studies in various cardiac patient cohorts confirm the effectiveness, and the safety, of dynamic resistance training [27,29,30,31,32] and showed its positive influence on numerous health and prognostic factors. (see Table 4).

#### 3.1.3. Limitations

So far, there is no clear scientific evidence in which form, intensity, and by whom monitoring and/or medical controls during ET should be performed. Recommendations are primarily based on expert opinions. High-intensity interval training (HIIT) is considered safe and effective [33,34,35,36] However, its use in early phase II of ebCR is currently controversial, especially in HFrEF patients [35,36]. The prognostic significance of muscular strength is poorly studied.

**Table 2:** Risk stratification regarding cardiac events during physical activity and exercise training in ebCR [2,15,37].

Low risk (all listed findings must apply)
Findings/Results of the graded exercise testing: No complex ventricular arrhythmias under increasing exercise and in the recovery phase. No angina or other significant symptoms (e.g., unusual shortness of breath, light-headedness, or dizziness) under increasing exercise or recovery or in the recovery phase Normal hemodynamics under increasing exercise and in the recovery phase (e.g., adequate heart rate increase and recovery, adequate increase in systolic blood pressure under increasing exercise, and decrease in the recovery phase). Exercise capacity ≥ 7 METs (≥1.8 watts/kg body weight). Other findings: No significant left ventricular dysfunction (EF ≥ 50%). uncomplicated course after myocardial infarction, bypass surgery or after elective coronary revascularization No complicated ventricular arrhythmias at rest No signs of heart failure No signs of residual ischemia No clinically relevant depression
Moderate risk (if diagnosed with one or more of the listed findings)
Findings/Results of the graded exercise testing: Onset of angina or other significant symptoms (e.g., unusual shortness of breath, light-headedness, or dizziness at higher exercise intensities (<7 METs (<1.4 watts/kg body weight)) during exercise testing Onset of mild or moderate silent ischemia during exercise testing or in the recovery phase (ST-segment depression < 2 mm from baseline) Exercise capacity <5 METs (<1.2 watts/kg body weight) Other findings: Moderately impaired left ventricular function (EF 40–49%).
High risk (if diagnosed with one or more of the listed findings)
Findings/Results of the graded exercise testing: Complex ventricular arrhythmias during exercise testing or in the recovery phase. Angina or other significant symptoms (e.g., unusual shortness of breath, light-headedness, or dizziness) at low exercise intensity (≤5 METs; 1.2 watts/kg body weight) during exercise testing and during the recovery phase Myocardial ischemia during exercise or in the recovery phase (ST-segment depression ≥ 2 mm) Pathological hemodynamics during exercise (e.g., chronotropic incompetence, flattening/decrease in systolic blood pressure during exercise) or in the recovery phase (e.g., severe post-exercise hypotension). Other findings: reduced left ventricular function (EF < 40%) condition after cardiac arrest or resuscitation complex arrhythmias at rest or during exercise myocardial infarction, cardiac surgery, or interventional coronary revascularization with consecutive shock or residual ischemia pulmonary hypertension condition after ICD implantation condition after CRT implantation condition after VAD implantation condition after heart transplantation (clinically stable) clinically relevant depression congenital heart disease and valvular heart disease require special individual cardiac assessment and consideration

**Table 3:** Moderate continuous aerobic endurance training (MCT): training parameters for exercise recommendations and exercise training control [4,5,7,8,38].

Parameter for Exercise Recommendations and Control	Recommendation Ranges	
Cycle Ergometer Test	Low to Moderate Intensity	Moderate to High Intensity
Percentage of peak heart rate achieved (% HR_peak_)	65–75% HR_peak_	75–85% HR_peak_
Percentage pf peak heart rate reserve ^1^ achieved (% HRR_peak_)	40–60% HRR_peak_	60–70% HHR_peak_
Exercise work load based on percentage of peak work load achieved in cycle ergometer test (watt_peak_)	40–60% watt_peak_	60–80% watt_peak_
Cardiopulmonary exercise testing ^2^		
Percentage of peak oxygen uptake achieved (% VO_2peak_)	40–60% VO_2peak_	60–80% VO_2peak_
Ventilatory threshold (VT1), respiratory compensation point (VT2)	from VT1 (55–70% VO_2peak_)	up to below VT2 (70–80% VO_2peak_)
Other parameters for exercise control ^3^		
Borg scale (6–20) for rating perceived exertion (RPE)	12–14 RPE	>15 RPE
Respiratory rate “speech rule” ^4^	the breathing during exercise should allow conversation	
Recommended exercise duration	from >5 up to 60 min	
Recommended exercise frequency	3–5 (7) days a week/most day of the week	

^1^ Calculation of the target exercise heart rate according to the Karvonen formula: Calculation example: target exercise intensity 40% of heart rate reserve; resting heart rate: 60 beats/min; peak heart rate achieved during exercise test = 120 beats/min; Target exercise heart rate = 60 + (120 − 60) × 0.4 = 84 beats/min. ^2^ Cardiopulmonary exercise testing: recommended especially in patients with chronic heart failure and congenital heart diseases. ^3^ in addition to other parameters, or when heart rate cannot be used for control (e.g., after heart transplantation, or VAD implantation). ^4^ Speech rule = moderate exertion should be performed without dyspnoea so that conversation is easily possible.

**Table 4:** Recommendations for setting up and performing resistance exercise within cardiac rehabilitation, modified from [2,3,4,5,7,8,16,39,40].

Training Stage	Training Objective	Training Method	Training Intensity	Number of Repetitions per Muscle Group	Training Volume
Initial stage (pre-training, familiarization)	Implementation of exercise: Learning and practicing the correct execution Improvement of self-perception and coordination	Dynamic	>30% 1-RM RPE ≤ 11	5–10	2–3 units per week, 1–3 sets per unit, 1–2 min rest between sets
Testing of muscular strength	Determination of the one-repetition maximum (1-RM)				
Improvement stage I	Improvement of local aerobic endurance, improvement of coordination	Dynamic	30–50% 1-RM, RPE 12–13	10–15	2–3 units per week, 1–3 sets per unit, 1–2 min rest between sets
Improvement stage II	Increase in muscle cross-sectional area (hypertrophy), improvement of coordination	Dynamic	40–60% 1-RM, RPE 14-1	8–15	2–3 units per week, 1–3 sets per unit, 1–2 min rest between sets
Improvement stage III	Increase in muscle cross-sectional area (hypertrophy), improvement of coordination	Dynamic	60–80% 1-RM, selected patients in good clinical condition	8–10	2–3 units per week, 1–3 sets per unit, 1–2 min rest between sets

1-RM = One Repetition Maximum, RPE = Rate of Perceived Exertion.

### 3.2. Physical Activity and Exercise Training in Patients with Coronary Artery Disease (CAD)

The following recommendations are based on most recent and topic-related scientific guidelines and by a semi-structured evaluation of scientific literature (classification: S2k; see Methods in Part 1).

#### 3.2.1. Recommendations

- All patients with stable CAD are recommended to participate in supervised individually adapted exercise training as part of ebCR. This applies to patients after acute coronary syndrome (ACS), unstable angina, STEMI, NSTEMI with/without PCI, and/or post bypass surgery (CABG). Refs. [3,4,5,7,8] (↑↑ 100%)
- After uncomplicated PCI, it is recommended to start an individually adapted and monitor-supervised ET as early as possible (from day 4 after PCI). Ref. [41] (↑↑ 100%)
- Early mobilization is recommended after ACS (STEMI, NSTEMI, unstable angina pectoris) and after CABG. In the case of an uncomplicated course, this should begin as early as 24–48 h after the event or post-op in the acute care hospital. Ref. [7] (↑↑ 100%)
- In the first 6–8 weeks (individual and symptom-dependent up to several months) after CABG, it is recommended to consider intervention-related limitations (e.g., wound healing disorders, pain after thoracotomy, sternum stability) when implementing and performing ET. (↑↑ 100%)
- In case of wound healing disorders with systemic inflammatory activation, causal therapy for the wound healing disorder is recommended to be given first, before starting any exercise intervention. Ref. [7] (↑↑ 100%)
- It is recommended (↑↑ 100%)
  - to introduce all patients with stable CAD to a supervised individually adapted and controlled continuous aerobic endurance training (MCT) as early as possible and to continue the MCT in the long term [4,5,7,8].
  - to start the MCT with low to moderate intensity:
  - In case of positive ischemia detection in the stress test, to establish the exercise intensity at a heart rate at least 10 beats below the ischemia threshold.
  - to control exercise intensity using subjective rate of perceived exertion (RPE) (Borg scale 11–14/20 RPE) [42] and/or breathing rate (should allow conversation, “speech rule”) [4,5,7,8].
  - to introduce stable patients with good exercise capacity to more intensive MCT during ebCR
  - to introduce supervised, and individually adapted, resistance training [30–60% 1-RM) in all stable patients with CAD (in operated patients with adequate sparing of the sternum) as early as possible and to continue the resistance training in the long term [3,4,5,7,8].
- In stable patients with good exercise capacity, high-intensity interval training (HIIT) may be considered in the long-term course. (↔ 100%)

#### 3.2.2. Scientific Evidence ebCR

There is well-documented evidence that participation of clinically stable CAD-patients in supervised, individualized ET leads to improved physical performance and symptom free exercise capacity [43,44,45], a reduction in angina symptoms, a positive impact on cardiovascular [46,47] and psychological risk factors [48], quality of life [46,48], and prognosis [10,11,12]. Aerobic endurance training positively affects the progression and symptomatology of CAD in multiple ways [8,19,49]. Endurance training (continuous and interval) is safe and effective for improving peak VO_2_ in CAD-patients [35,43,44,45,50,51]. An increase in exercise capacity during CR is associated with a reduction in all-cause mortality [45]. Combined endurance and resistance training shows greater effectiveness on body composition [52], muscle strength [30,52], peak VO_2_, and mobility [30] compared with endurance training alone. In previously inactive individuals, increasing activity and continuing a previously active lifestyle after manifestation of CAD is associated with a reduction in all-cause mortality [53,54].

#### 3.2.3. Limitations

The efficacy and safety of the short-term [14] interval training (including high-intensity interval training (HIIT)) in CAD patients with preserved left ventricular pump function well studied, but most studies were conducted in patients at a later stage and not during phase II CR. Long-term results are missing

### 3.3. Physical Activity and Exercise Training in Patients with Congestive Heart Failure (CHF)

The following recommendations are based on the most recent and topic-related scientific guidelines and by a semi-structured evaluation of scientific literature (classification: S2k; see Methods in Part 1).

#### 3.3.1. Recommendations

- It is recommended (↑↑ 100%)
  - to perform a thorough risk evaluation and diagnostics, and check baseline conditions before starting the ET:
  - to check and exclude contraindications before starting the ET [7,55,56]
  - to exclude an exercise induced myocardial ischemia before starting the ET [7,55,56].
  - to determine the indication for ICD before the start of the ET [7,56]
  - to check and exclude dehydration and hypervolemia, and perform regular weight control as an integral part of an exercise session [7,56].
  - to perform an exercise test (preferably cardiopulmonary exercise test with symptom-limited exercise over 8–12 min) to assess exercise capacity and exercise-related parameters for individualized exercise prescription [5,6,7,56].
  - to assess functional capacity with the 6-min walk test and use the results (6-min walking distance, % peak HR, RPE, breathing rate) to support the assessment of exercise tolerance and for individually adapted exercise prescription [6].
- After a long period of bedrest, in clearly deconditioned patients, in cachexia, or after clinical instability, an individualized stepwise mobilization (including light exercises to improve flexibility and muscular strength) is suggested to be performed as early as possible in preparation for exercise training. If necessary, this should be performed while the patient is still in the hospital. (↑ 100%)
- Supervised individually adapted continuous aerobic endurance training (MCT) is recommended as a basis exercise program for all patient with stable chronic systolic (HFrEF), diastolic (HFpEF), and mid-range (HFmrEF) heart failure of any age group during ebCR [5,6,7,8,55,57]. (↑↑ 100%)
- It is recommended (↑↑ 100%)
  - to start the MCT with low to moderate intensity [5,6]
  - to introduce stable CHF patients with good exercise tolerance to more intensive MCT during ebCR.
  - to increase exercise duration gradually up to 2060 min, 3–5 days per week starting with 5–10 min.
  - to start the MCT at a low intensity (i.e., 40–50% of peak VO2) and to keep the duration of MCT short (5–10 min, 2 times per week) in deconditioned CHF-patients with poor exercise capacity or patients with severely reduced LV function.
  - to prolong MCT duration and increase frequency if the deconditioned CHI patient tolerates the low intensity endurance exercise well.
- Alternatively, or complementarily MCT, aerobic interval training (IT) with 20–30 s of load at 85% to <100% peak watt alternating with 40–60 s of recovery, may be considered in all CHF-patients [7]. (↔ 100%)
- In the long-term course (phase III rehabilitation), high-intensity interval training (HIIT), with longer intensive exercise phases (e.g., 1–4 min at 85–95% of HRpeak) alternating with recovery of moderate intensity (1–3 min at 65% of HRpeak), may be considered in selected, stable CHF-patients [5,6]. (↔ 100%)
- Complementary to the aerobic exercise it is recommended: (↑↑ 100%)
  - to introduce supervised low to moderate intensity resistance training (30–60% 1RM) to all stable CHF-patient [5,6,7].
  - to include individually adapted exercises to improve coordination (especially sensorimotor training and balance training) and flexibility, as well as gait training, into the exercise program.
  - to include an inspiratory muscle training (IMT) if necessary, (i.g. in patients with respiratory muscle weakness) [5,6,58,59].
- If necessary, functional electrical stimulation may be considered (e.g., in significantly deconditioned patients) [60]. (↔ 100%)

#### 3.3.2. Scientific Evidence

There is well-documented evidence that individually adapted exercise training is effective to prevent muscular deconditioning with all its negative implications on activities of daily living and to improve exercise capacity, mobility [61,62], and health-related quality of life in heart failure patients [24,61,62,63]. An increase in exercise capacity during ebCR is associated with a reduction in all-cause mortality and hospitalisation [64]. Moderate continuous aerobic endurance training (MCT) is safe and effective to improve exercise capacity in HFrEF-patients [13,32,43,65] and leads to several beneficial clinical effects [20,21,22,23,66,67,68].

Interval training (IT) with short bursts (20–30 s) of intense exercise is well tolerated [28,69,70] and leads to comparable improvements in exercise capacity and mobility as MCT [28,69,70]. Low to moderate intensity resistance training in HFrEF-patients is safe [32], and does not affect LV systolic function negatively [32]. It is effective to improve muscular strength [71], reduce disease related loss of muscle mass and strength [71], as well as increase mobility [72], exercise capacity, [32] and quality of life [71]. Combined endurance and resistance training shows greater effectiveness on exercise capacity and mobility compared with either intervention alone.

In patients with HFpEF, aerobic endurance training (MCT or interval) and resistance training improve LV diastolic function [21,73,74,75,76,77], exercise capacity [73,74,75,76,77,78,79], and quality of life [75,77,78,80]. High level of physical activity is associated with reduced all-cause mortality, cardiovascular mortality, and hospitalization rate, due to decompensated HFpEF, compared with low activity level [81].

In ebCR, patients with pulmonary arterial hypertension (PAH) and chronic thromboembolic pulmonary hypertension (CTEPH) represent a very special entity. However, low dose exercise training and specialized rehabilitation in these patients has been well established [82]. The potential role of rehabilitation, after pulmonary endarterectomy (PEA) in CTEPH patients, has only been studied in a small, nonrandomised retrospective study [83]. Another small, but prospective and randomised, study showed that the combined approach of balloon pulmonary angioplasty (BPA) and a structured rehabilitation programme was more successful than BPA alone [84].

Based on these studies, a carefully monitored, low-dose rehabilitation programme after surgical or BPA treatment of CTEPH patients might be considered standard of care [85]. Large-scale randomised trials and studies on mechanisms of the effect of exercise training after pulmonary endarterectomy and balloon angioplasty are needed.

#### 3.3.3. Limitations

Large well-powered ebCR RCTs in heart failure patients are rare and results of meta-analysis dominated by one large trial [86]. Moreover, available trials show a considerable heterogeneity in study design, and ebCR intervention provided. In addition, the patient populations varies substantially with regard to age, disease history and aetiology, as well as comorbidity and cardiorespiratory fitness [24]. For HFrEF and an LV-EF ≤40%, there is no evidence for a reduction in all-cause mortality, cardiac mortality, or rehospitalization as a result of ebCR [24,61,62,63].

The effectiveness of high-intensity interval training (HIIT) is meanwhile well documented but the results are conflicting [87,88,89]. A superiority of HIIT to improve exercise capacity, end-diastolic diameter, or left ventricular ejection fraction, compared to MCT, could not be confirmed [36]. Furthermore, long-term outcomes are unclear, and a trend toward higher rates of adverse events during follow-up after HIIT has been observed [36].

### 3.4. Physical Activity and Exercise Training in Patients with Surgical or Interventional Heart Valve Therapy

The following recommendations are based on the most recent and topic-related scientific guidelines and by a semi-structured evaluation of scientific literature (classification: S2k; see Methods in Part 1).

#### 3.4.1. Recommendations

- In patients after surgical or interventional heart valve replacement (including corrective procedures) the participation in supervised structured and individualized aerobic endurance and moderate dynamic resistance training during ebCR is recommended. (↑↑ 100%)
- It is recommended (↑↑ 100%)
  - to perform the ET within the first postoperative week in a medically supervised setting.
  - to increase the exercise intensity and volume, adjusted individually and gradually.
  - to avoid inadequate shear, compression, and extension loads on the thoracic skeleton and sternum within the first 6–8 postoperative weeks after thoracotomy.
  - to include individually adapted exercises to improve coordination (especially sensorimotor training and balance training) and flexibility, as well as gait training, into the exercise program.
  - to avoid all contact sports or other injury-prone exercise in patients on oral anticoagulation

#### 3.4.2. Scientific Evidence

Results from a large cohort study show participation in ebCR to be associated with decreased 1-year cumulative hospitalization, and mortality risk, after valve surgery [90]. As a result of exercise training (mainly aerobic endurance training), improvements in physical performance, exercise capacity, mobility, and muscular strength have been reported [91,92,93,94,95,96].

#### 3.4.3. Limitations

There is a lack of evidence for the impact of exercise training after surgical or interventional heart valve correction [95]. The few available studies, mostly small RCT and cohort studies report results from heterogenic ebCR measures, with regard to patient profile [95], type of intervention [97], as well as ebCR duration [94,97].

Qualitatively sufficient evidence-based data on adverse events, mortality, quality of life, symptomatology, and reversible left ventricular “remodeling” are not yet available [95]. High quality RTC, evaluating the impact of exercise training after surgical or interventional heart valve correction, are missing. For patients after MitraClip, percutaneous mitral, or tricuspid valve replacement, no studies on physical training are available yet.

### 3.5. Physical Activity and Exercise Training in Patients in Patients after Implantation of Cardioverter-Defibrillator (ICD), Resynchronisation System (CRT), and Patients with a Wearable Cardioverter-Defibrillator (WCD)

The following recommendations are based on the most recent and topic-related scientific guidelines and by a semi-structured evaluation of scientific literature (classification: S2k; see Methods in Part 1).

#### 3.5.1. Recommendations

- For patients after ICD and CRT implantation, it is recommended (↑↑ 100%)
  - to perform the ET initially ECG-monitored and under medical supervision in order to define appropriate exercise prescription.
  - to consider the underlying cardiac disease, the clinical condition of the patient and the ICD and CRT programming when developing individual training recommendations.
  - to determine the peak heart rate allowed to be achieved during ET clearly (at least 10–20 beats) below the programmed detection rate of the ICD.
  - to use heart rate monitor during independent/non supervised ET
  - to avoid sports/exercises with increased situational danger in case of shock delivery, where there is a particular danger when the person is alone (e.g., diving; swimming should only be practiced in shallow water and with the protection of a possible helper).
  - to strictly avoid sports with increased risk (e.g., climbing, motor sports, parachuting, paragliding, martial arts).
  - to avoid all sports/exercises with intensive shoulder-arm movements on the side where the ICD, CRT is implanted or with mechanical loads on the torso area where the ICD is implanted.
  - to additionally apply the disease-specific recommendations in patients after ICD, CRT implantation in HFrEF-patients
  - to define the individual exercise recommendations in patients with WCD primarily based on the underlying disease and, in addition, analogously to the recommendations for ICD-patients.

#### 3.5.2. Scientific Evidence

Results of controlled trials have demonstrated the safety and effectiveness of supervised exercise training after CRT/ICD implantation for various underlying cardiac conditions with both primary and secondary prophylactic implantation indications [98,99,100,101,102,103,104,105]. In heterogeneous patient populations, a randomized trial even described a reduction in shock delivery in the exercise group [103,105]. In contrast, registry data show a selectively increased shock rate in young patients with intensive recreational or competitive sports activity, but without increased mortality [105,106].

#### 3.5.3. Limitations

These partly underpowered studies integrate a very heterogeneous patient population with respect to the underlying disease and the exercise interventions used show a large variance. In addition to these studies in consistently heterogeneous patient populations, the data are supplemented by exercise training studies in heart failure patients and patients with impaired pump function only

### 3.6. Physical Activity and Exercise Training in Patients with Ventricular Assist Device (VAD)

The following recommendations are based on most recent and topic-related scientific guidelines and by a semi-structured evaluation of scientific literature (classification: S2k; see Methods in Part 1).

#### 3.6.1. Recommendations

- For hemodynamically stable VAD-patients, the participation in medically supervised and individually adapted ET is suggested [107,108,109,110]. (↑ 100%)
- In patients with newly implanted VAD-system it is suggested to start the training primarily depending on the individual clinical condition and disease progression. (↑ 100%)
- It is recommended (↑↑ 100%)
  - that all therapists (physicians, physiotherapist, exercise therapist) entrusted with ET in VAD-patients demonstrate in-depth knowledge in this field, based on current VAD-guidelines [108,110]
  - that the ET is led by experienced, well-trained exercise therapists, who are familiar with the VAD-systems used, safety aspects, and special emergency management [108,110].
- It is recommended (↑↑ 100%)
  - to observe the following discontinuation criteria before starting any exercise session as well as during the exercise training after VAD implantation [108,109,110]:
  - Pump flow reduction below 3 L/min
  - Increase in the energy demand of the pump in watt (CAVE: thrombus!!)
  - decrease in peripheral O_2_ saturation <90% (pulse oximeter)
  - Bleeding (e.g., nosebleed)
  - to pay attention to the following safety aspects before starting exercise [108,109,110]:
  - control of rechargeable batteries
  - attention to the driveline length and position
  - positioning of the external equipment
  - to integrate education and training of patients in the handling of the VAD-system and the safety aspects during physical activity and exercise training, but also during exposure to exertion in everyday life, leisure time and, if necessary, at work [107,108,110].
- Abrupt changes in position (e.g., movement from sitting to standing with a rapid shift in blood volume) generally are not recommended when performing any form of exercise. Refs. [104,106] (↓↓ 100%)
- All activities that could lead to an uncontrolled, inadequate strain on the system are (e.g., contact sports) are not recommended. Refs. [108,110] (↓↓ 100%)
- It is recommended (↑↑ 100%)
  - to introduce supervised individually adapted low to moderate MCT or IT (20–30 s of load alternating with 40–60 s recovery) as a basis exercise program for VAD-patient [108,110].
  - to use the BORG scale (starting with RPE ≤13 and possible increase to RPE ≤15) and the respiratory rate (speaking rule) to control exercise intensity [107,108,110].
  - to define the excise intensity at 40–60% of peak VO_2_, at 40–50% of peak Watt, or close to the ventilatory threshold (VT1), if available [107,108,110].
- Individually adapted low to moderate intensity resistance training with a low isometric component may be considered as a complementary exercise mode to the aerobic endurance training. Refs. [107,108,110] (↔ 100%)
- In respect to resistance training, it is recommended to take special care in the selection of exercises and training equipment [107,108,110]. (↑↑ 100%)
- It is suggested, to focus the resistance training on exercises for the muscles of the lower extremities [107,108,110]. (↑ 100%)
- All exercises for the abdominal and back muscles are not recommended [108,110]. (↓↓ 100%)
- It is recommended to take special care in the selection and performance of all exercises of the muscles of the upper extremities and shoulder girdle [108,110]. (↑↑ 100%)
- If necessary, (i.e., in patients with respiratory muscle weakness) an inspiratory muscle training (IMT) is recommended [108,110]. (↑↑ 100%)

#### 3.6.2. Scientific Evidence

Results of a recent meta-analysis and systematic reviews show a positive effect of ebCR on exercise capacity, mobility, and quality of life [111,112,113,114]. However, these studies provide evidence that VAD-patients may benefit, at least in the short term, from individually adapted endurance training [115,116,117,118,119,120,121], resulting in improvements in exercise capacity [116,121] mobility [116,121], quality of life [115,121], and pulmonal capacity [121]. Four studies report experience with resistance training [115,118,119,122] and confirm the effectiveness, feasibility, and good tolerance in this patient group. Relevant contraindications to exercise-based interventions specifically for VAD-patients have not been published [110,123]. Long-term effects of exercise therapy measures are not yet available [110,124,125]. The results of a retrospective analysis show ebCR participation to be associated with lower one-year hospitalization risk and one-year mortality risk [126]. In summary, integrating the experience gained in chronic heart failure [5,6], the results allow cautious recommendations for ET in VAD-patients [107,108,110,123,127,128].

#### 3.6.3. Limitations

The available evidence does not allow reliable conclusions about the safety [111,112,113,114,123,125,129] and effectiveness of exercise training in VAD-patients [111,112,113,114,124,125,130]. Results are available from a few retrospective analyses [118,119,120,131], controlled or comparative studies [117], and very small randomized controlled trials [115,116,121] reporting experiences and outcomes based on a very heterogeneous patient population.

### 3.7. Physical Activity and Exercise Training in Patients after Heart Transplantation (HTX)

The following recommendations are based on most recent and topic-related scientific guidelines and by a semi-structured evaluation of scientific literature (classification: S2k; see Methods in Part 1).

#### 3.7.1. Recommendations

- It is recommended (↑↑ 100%)
  - to introduce a supervised individually adapted continuous aerobic endurance training (MCT) as early as possible (2nd-3rd week postoperatively) [5,132,133].
  - to start a moderate individually adapted dynamic resistance training (50–60% 1-RM) as early as possible postoperatively, latest during the CR [5,132,133].
  - to perform all exercise measures during ebCR with adequate sparing of the sternum.
- It is suggested (↑ 100%)
  - to perform a graded exercise test in small increments of 5–15 watt, to assess potential exercise intensity, earliest from the 3rd week postoperatively onward [134,135].
  - to start the aerobic endurance training with the intensity set at <50% of the peak VO_2_ or 10% below the ventilatory threshold (VT1) [5].
  - to use the perceived rate of exertion (BORG scale 11–14/20 RPE) and/or the respiratory rate (“speaking rule”) to control and adapt exercise intensity.
  - to introduce stable patients with good exercise capacity to more intensive endurance training in the long-term course (possibly also as high intensity interval training) [136].
- It is recommended to reduce or (in the case of cortisone bolus therapy) completely discontinue all exercise activities during rejection episodes, depending on their severity [133]. (↑↑ 100%)
- Ischemia diagnosis is suggested before restarting ET in the late stage after HTX. (↑ 100%)
- It is recommended to continue the ET in the long term (lifelong) [5,132,133]. (↑↑ 100%)

#### 3.7.2. Scientific Evidence

The results from a meta-analysis confirm the effectiveness of ebCR after HTX to improve exercise capacity [137]. Moderate continuous aerobic endurance training (MCT) is effective to improve exercise capacity [120,136,138,139,140,141], heart rate recovery [141], skeletal muscle oxidative capacity [140], blood pressure control [25], endothelial function [26,27,142] quality of life [120,143,144,145], and to increase peak heart rate [140]. High-intensity interval training (HIIT) is feasible after HTX and effective to improve exercise capacity [141,146,147,148,149,150], blood pressure control [141], endothelial function [151], quality of life [145,150], heart rate recovery [141], increase peak heart rate [141], and decrease anxiety and depression [150,152]. Moderate resistance training (50% 1-RM) improves muscle strength [138] and counteracts the negative effects of immunosuppressive therapy on muscle and bone metabolism [31,153].

#### 3.7.3. Limitations

First indications of a protective role of ebCR immediately after transplantation provided by the results of a smaller retrospective study, show a significant association between the number of CR-units completed in the first 90 days after HTX with survival [154]. Currently, there are no studies available demonstrating a beneficial long-term effect of exercise interventions of any kind on the prognosis and clinical course of HTX patients [132,155]. In addition, there are no data evaluating ebCR in the subgroup of HTX patients with systemic diseases, such as neuromuscular disorders, in whom exercise training would be most important to reduce their muscular weakness.

Cardiac allograft vasculopathy (CAV) is a progressive form of coronary artery disease (CAD) leading to increased morbidity and mortality in patients after HTX. In a randomised IVUS analysis of 43 HTX patients, participation in HIIT over one year sig-nificantly reduced the increase in coronary atheroma volume as compared to a usual care group that had not received any special recommendation regarding exercise intensity [156]. However, the impact of different exercise training modalities on the progression of CAV requires further investigation [157].

### 3.8. Physical Activity and Exercise Training in Patients Peripheral Artery Disease (PAD)

The following recommendations are based on the most recent and topic-related scientific guidelines and by a semi-structured evaluation of scientific literature (classification: S2k; see Methods in Part 1).

#### 3.8.1. Recommendations

- All patients with PAD in the Fontaine stages I (after surgical or interventional therapy), IIa, and IIb, are recommended to be introduced to an individually adapted and supervised ET as early as possible, taking into account their comorbidities [158,159,160,161,162]. (↑↑ 100%)
- After surgical or interventional revascularization, it is recommended. (↑↑ 100%)
  - to pay attention to procedure-related limitations (e.g., wound healing disorders, lymphedema, neuropathic pain) during the first weeks of ET.
  - to carry out suitable physiotherapeutic measures and provide appropriate auxiliary aids to support the implementation of the ET d
  - uring ebCR.
- In symptomatic and asymptomatic patients of any age group, conservatively treated or after interventional/surgical revascularization (PTA, TEA, bypass surgery) are recommended [158,159,160,161,162]. (↑↑ 100%)
  - to perform interval walking on a treadmill or on a firm flat surface.
  - to perform the interval walking at individual pace until patients reach mild to moderate claudication pain, and then rest (2–3 min) until the pain subsides completely in symptomatic patients.
  - to pay attention to new cardiac symptoms (e.g., angina pectoris, dyspnoea, pretibial oedema, hypertension, tachycardia) during the course of the disease, especially in the case of improved ambulation and walking distance in the case of cardiac comorbidity (e.g., CAD, CHF, arrythmias) [4].
  - to perform exercises to improve balance, gait quality and to increase flexibility as well as the local aerobic endurance capacity
- As a complement to the interval walking, it may be considered. (↔ 100%)
  - to perform interval training on a bicycle ergometer or arm ergometer training [163].
  - to perform low to moderate intensity dynamic resistance training (30–60% of 1-RM).

#### 3.8.2. Scientific Evidence

There is well documented evidence for the effectiveness of interval walking training [164,165,166,167,168,169]. Performed on a treadmill or on a firm flat surface it is effective to improve walking ability [169], to prolong maximum walking time [169], pain-free walking distance [165,169,170], maximum walking distance [165,169,170], and 6-min walking distance [165,166], as well as to improve exercise capacity [166,171] and quality of life [166,167,168,169] and Walking Impairment Questionnaire Score (WIQ score) [165,166]. The importance of exercise training has also been demonstrated after successful revascularization [172,173,174]. Compared with revascularization alone or interval waking training alone, the combination of both measures is associated with a significant improvement in maximal walking distance [173,174] and reduced risk of recurrent revascularization or amputation [173]. Documented effects of resistance training in PAD-patients are increased 6-min walking distance [175,176,177], walking performance [176,177], maximal walking time [176,177], and muscle strength [175,176,177], as well as improvement in quality of life [162] and WIQ score [162].

#### 3.8.3. Limitations

Despite beneficial effects on morbidity, the prognostic impact of supervised interval walking has been poorly studied [169,178]. Other exercise interventions have not been adequately investigated [163].

### 3.9. Physical Activity and Exercise Training in Patients with Myocarditis

The following recommendations are based on the most recent and topic-related scientific guidelines and by a semi-structured evaluation of scientific literature (classification: S2k; see Methods in Part 1).

#### 3.9.1. Recommendations

- Exercise training is not recommended in cases of biopsy-confirmed myocarditis or clinically high-grade suspicion of acute or chronically active myocarditis. (↓↓ 100%)
- In case of biopsy-confirmed myocarditis or clinically high-grade suspicion of acute or chronically active myocarditis, performance-oriented or competitive sports activities are not recommended for at least 3–6 months. (↓↓ 100%)
- Before starting any ET, it is recommended to perform a careful cardiac risk stratification, including Holter-ECG (see Part 1). (↑↑ 100%)
- In patients with heart failure as a possible or proven long-term consequence of myocarditis, the introduction to an individually adapted exercise training is recommended in case of a stable course of the disease and guideline-compliant drug therapy. (↑↑ 100%)

#### 3.9.2. Scientific Evidence

Biopsy-proven or clinically highly probable acute or active myocarditis is considered a frequent cause of sports-associated sudden cardiac death [179,180]. Therefore, any exercise training is strictly contraindicated in acute or active myocarditis [181]. Consensus recommendations advise an exercise break of at least 3–6 months with subsequent evaluation of the cardiac situation even after healing of the myocarditis [181,182]. The prognostic significance of residual myocardial fibrosis documented by cardiac MRI with regard to exercise training is still unclear [181]. Due to an arrythmogenous risk potential, at least regular cardiological follow-up is recommended in such cases [183,184].

#### 3.9.3. Limitations

To date, no specific studies on exercise training as a sole intervention or a component of CR are available for patients after myocarditis. (See part 1) [185,186].

### 3.10. Physical Activity and Exercise Training in Patients with Congenital Heart Disease (ACHD)

The following recommendations are based on the most recent and topic-related scientific guidelines and by a semi-structured evaluation of scientific literature (classification: S2k; see Methods in Part 1).

#### 3.10.1. Recommendations

- It is recommended (↑↑ 100%)
  - to give advices for PA and ET in ACHD-patients based on results of thorough risk stratification, including stress test [187,188,189,190,191,192,193,194,195,196,197].
  - to give special attention to stress-induced (malignant arrhythmias, arterial hypertension, aortic dissection, myocardial ischemia, exacerbation of heart failure, increase in cyanosis, syncope, and sudden cardiac death) [187,198].
  - to provide every ACHD-patient with counselling regarding the preventive importance and individual options for PA and ET [189,190,191,194,197].
- Asymptomatic patients with no or only mild residual findings and no activity limitations are recommended to participate in all individually adapted exercise intervention available/offered during CR. Refs. [189,190,194,197] (↑↑ 100%)
- In patients with significant residual findings, with complex heart defects or after palliative corrected heart defects (e.g., Fontan patients, corrected transposition of the great arteries (cc-TGA), systemic right ventricle) it is suggested to introduce them to supervised, individually adapted exercise-based interventions with a low dynamic and static load, if their clinical condition permits. Refs. [187,192,193] (↑ 100%)
- It is suggested to emphasize the exercise training on the lower extremities. Refs. [187,192,193,194,199] (↑ 100%)
- If necessary, (i.g. in patients with respiratory muscle weakness) an inspiratory muscle training (IMT) is recommended. (↑↑ 100%)
- In patients who have recently undergone sternotomy, those on anticoagulation therapy, and/or have undergone pacemaker and ICD implantation, it is recommended to follow and take into account the relevant recommendations for these conditions in planning and implementation of exercise training. (↑↑ 100%)

#### 3.10.2. Scientific Evidence

ACHD-patients benefit at least in the short term from individually adapted endurance training [200,201,202,203,204,205,206,207,208,209] and that significant improvements in exercise capacity [199,202,205], oxygen pulse [201], blood pressure [200,207], functional capacity [198,202], activity level [201,206], and quality of life [202,206] can be achieved. No negative effects of exercise on “remodeling” [210] or systolic and diastolic ventricular function (in tetralogy of Fallot) were observed [211].

#### 3.10.3. Limitations

Evidence on the feasibility, efficacy, and safety of physical activity and exercise training in ACHD is limited [188,189,190,198]. Results are available from few cohorts [202,205,206], controlled [199,200,203], and randomized trials [201,204,207,208,209], reporting in particular on patients with significant residual findings and complex cardiac defects.

One study reported results of strength endurance training in Fontan patients, which was well tolerated and led to improvement in muscle strength, muscle mass, and physical performance [199].

### 3.11. Psychological Interventions

The following recommendations are based on the meta-analysis MAPSY [212] in addition to other recently published meta-analyses and trials. (classification: S3; see Methods in Part 1).

The most prominent concept in CR in western countries is exercise-based CR (ebCR), which additionally may include education, support of individual lifestyle changes, optimisation of evidence-based medication, and various psychological interventions, then defined as “multi-component” or “multimodal rehabilitation”. In German speaking countries, psychological interventions are an integral part of cardiac rehabilitation [213]. Therapeutic goals, that should be agreed upon between the patient and the therapeutic team, encompass coping with illness, especially the reduction in anxiety and depressive symptoms, adherence to medical and non-pharmacological therapy, and distress management.

Several previous meta-analyses on psychological interventions in CVD patients have demonstrated beneficial effects on symptoms of depression, anxiety, distress and on quality of life (QoL) [214,215,216,217,218] (see Table 5). However, effects on cardiovascular morbidity, cardiovascular and total mortality were inconclusive, with predominantly older studies before 1995 showing positive effects. In addition, these meta-analyses could not control for the effects of modern ebCR, pharmacotherapy and coronary interventions, and effects on morbidity/mortality might be overestimated. Complementary meta-analyses on ebCR also reported at least short time positive effects on depressive and anxious symptoms, and “mental health” [217,219,220,221,222], but due to methodological limitations they could not rule out the specific contribution of psychological interventions on these effects. For that reason, we performed a systematic review in order to evaluate the current efficacy of additional, well defined psychological interventions compared with ebCR alone on depression, anxiety, QoL, cardiovascular morbidity, cardiovascular mortality, and total mortality in CVD patients [212]. Based on a comprehensive evaluation of the evidence, the following recommendations have been formulated.

#### 3.11.1. Recommendations

- Psychosocial factors (e.g., depressive symptoms, anxiety, stress, motivation, self-efficacy) are recommended to be detected at the beginning of cardiac rehabilitation and to be evaluated whether they need to be treated (“Screening”). (↑↑, 4, 100%)
- This screening is suggested to be supported by specific and validated tools such as questionnaires. (↑, 4, 100%)
- Based on this screening procedure it is recommended to deliver appropriate psychological interventions and support after a shared decision making. (↑↑, 4, 100%)
- Non-specific psychological interventions to all patients in CR without individual indication are not recommended. (↓↓, 4, 100%)
- Mental disorders, according to ICD-10, are recommended to be diagnosed and treated according to guidelines taking into account cardiovascular comorbidities. (↑↑, 1-, 100%)
- Mental disorders according to ICD-10 are not recommended to be treated only by psychological interventions to improve health behavior, coping with the disease and distress management. (↓↓, 1, 100%)
- Psychological interventions are suggested to be performed by qualified physicians or psychologists. (↑, 4, 100%)

#### 3.11.2. Scientific Evidence

Meta-analyses, until 2018, exhibit that psychological interventions are able to improve quality of life, depressive symptoms, and anxiety in patients with cardiovascular disease (CVD). However, significant effects on cardiovacular morbitiy and mortality were heterogeneous (Table 5).

As a conclusion of the studies displayed in Table 5, there is evidence for positive effects of psychological interventions not only on subjective health perception, but also on clinical endpoints. With the meta-analyses published until 2018, it is not possible, however, to discriminate an incremental add-on effect of a “multi-component” cardiac rehabilitation, including psychological interventions, as compared to exercise based cardiac rehabilitation without psychological interventions.

This was the rationale for a systematic literature review with meta-analysis, displayed in Table 6 [208]. The main research question was, whether it is possible to improve clinical endpoints of ebCR by adding psychological interventions as compared to a control group of ebCR without psychological intervention [212]. All criteria were defined using the PICO system:

Study design: randomised controlled studies (RCT) and controlled cohort studies (CCT), published after 1995

Population (P): ≥18 years, men and women with coronary heart disease (CAD, ACS, elective PCI, CABG, chronic coronary syndrome) and patients with congestive heart failure (CHF)

Intervention (I): Cardiac Rehabilitation (“exercise based CR”), including unspecific psychological support and/or transfer of knowledge and in addition at least one of the following specific psychological interventions, delivered by therapy specialists:

- psychologically supported lifestyle change
- distress management
- Combination of psychologically supported lifestyle change and distress management

Control-Intervention (C): “exercise based cardiac rehabilitation”, including unspecific psychological support and/or transfer of knowledge but without any specific psychological interventions

Outcomes (O): depressive symptoms, anxiety, quality of life (QoL), cardiovascular morbidity and mortality, total mortality

Systematic literature review covered the time from January 1995 to October 2017 including 17 RCTs and 3 CCTs with a total of 4.450 patients (CHD 88.5%, CHF 11.5%). The sample size varied from 59 to 1.127 patients and the follow-up interval ranging between the required minimum 6 months 5 years. Patient populations were predominantly mixed (ACS, CCS, elective PCI, CABG) and one study only included patients with CHF. Psychological characteristics at entry such as “poor motivation”, “psychosocial stress” or “depression” were specified in three studies, only. Another three studies excluded patients with “psychosocial problems” or “mental disorders”. In general, methodological quality of the studies was poor to moderate. Results of this meta-analysis are displayed in Table 6.

Based on heterogeneous studies, this meta-analysis did not show a significant incremental positive effect of psychological interventions when added to “exercise-based rehabilitation” (SMD = −0.13; 95% CI (−0.30 −0.05). Regarding specific interventions for “distress management” and “lifestyle change” there was a positive trend towards reduction in depressive symptoms [212]. Concerning the clinical endpoints, morbidity, mortality, and quality of life, meta-analysis could not be perfomed due to small sample size and heterogeneity of data. In three studies, investigating a similar “disstress management” over a one year period and following patients for an additional 5 years, there was a homogeneous trend towards reduction in cardiovascular morbidity (non-fatal MI, unstable angina, stroke, TIA, PCI, CABG, and peripheral revascularisation) that just failed to be significant (RR 0.74; 95% CI (0.51–1.07) [212].

Psychological interventions specifically targeting lifestyle change and distress in addition to ebCR may have an additional impact on symptoms of depression and cardiovascular events. However, an additional impact on anxiety, QoL, and cardiovascular mortality could not be confirmed by our meta-analysis [212]. After this publication, no meta-analyses with comparable methodological approach was published. Hence, taken together with favourable effects from stand-alone psychological interventions in cardiac patients [214,215,216,217,218]; all studies displayed in Table 5), evidence still supports the recommendation given by the European Society of Cardiology prevention and rehabilitation [9] that ‘multimodal interventions’ such as ebCR should include distinct psychological interventions, adjusted to the needs of the individual patient.

#### 3.11.3. Limitations

Empirical evidence supports that psychological risk factors such as disstress, anxiety, and depressive symptoms negatively affect quality of life, coping with the disease, and the individual health behaviour [224]. However, due to small sample size, heterogeneity of the psychological interventions applied, not well defined control group, short follow-up period, and older studies without modern cardiac therapy, it is not possible by now to define the incremental positive effect of additional psychological support during exercise based cardiac rehabilitation [212]. In addition, there is considerable uncertainty regarding which specific psychological interventions may work best for whom and under which conditions [212]. Psychological interventions during ebCR might still have a significant impact if they are delivered specifically and individually to the patient’s need by qualified physicians or psychologists. In terms of evidence based medicine, this has to be further investigated by appropriate studies.

##### Patient Education

Patient education for secondary prevention of cardiovascular diseases is an essential part of a comprehensive cardiac rehabilitation (CR) and aims to promote patient self-management and empowerment. Educational interventions have been shown to increase patients’ knowledge, as well as behavior changes regarding physical activity, dietary habits, and smoking cessation. The content, dose, and mode of delivery varied substantially between the included interventions, and there is still uncertainty about the optimal approach. Therefore, adequate education strategies for CR especially with regard to therapy adherence are essential. In addition, the content should be critically scrutinized and prioritized in terms of practical relevance and action-orientation, avoiding excessive demands on the patients.

### 3.12. Education to Strengthen Motivation and Adherence in General

The following recommendations are based on the most recent and topic-related scientific guidelines and by a semi-structured evaluation of scientific literature (classification: S2k; see Methods in Part 1).

#### 3.12.1. Recommendations

- Education is recommended to support motivation and personal responsibility to take part in therapeutic activities during CR and to promote coping with the disease. (↑↑, 100%)
- Education is recommended to use cognitive-behavioral techniques (such as goal setting, planning, self-monitoring, feedback, and motivational interviewing) to facilitate changes in behaviour. (↑↑, 100%)
- Education is suggested to implement a structured group approach including different patient-centered methods to actively involve the patient in order to deliver knowledge and skills tailored to the specific situation of the individual. (↑, 100%)

#### 3.12.2. Scientific Evidence

Adherence to a healthy lifestyle is crucial for the long-term effects of cardiac rehabilitation (CR) [9,225,226,227,228]. Supporting awareness, counseling, and educating the patients on an individual basis can increase knowledge, motivation, and skills and change the attitude towards the disease, thus enhancing adherence significantly [226,229,230,231,232,233,234].

A systematic review with meta-analysis including lifestyle modification programs for patients with coronary heart disease (23 studies, n = 11.085 patients [229]) exhibited during follow-up periods up to 60 months as compared to the control group:

- reduction in total mortality (OR 1.34, 95% CI 1.10–1.64; *p* = 0.003) in 6 RCTs; n = 6.270 patients,
- reduction in cardiac mortality (OR 1.48, 95% CI 1.17–1.88; *p* = 0.001) in 5 RCTs; n = 5.237 patients
- reduction in myocardial reinfarction and rehospitalisation (OR 1.35, 95% CI 1.17–1.55; *p* < 0.001) in 8 RCTs; n = 6.479 patients.

An odds ratio >1.0 corresponds to a higher event rate in the control group.

In addition, there were significant favorable effects on other cardiovascular risk factors (blood pressure, BMI, physical activity, and nutrition).

Another systematic review with meta-analysis including multimodal interventions to increase adherence to secondary preventive pharmacotherapy in patients with coronary artery disease [232] revealed significant positive results of these interventions (OR 1.52, 95% CI 1.25–1.86; *p* < 0.001; 16 RCTs; n = 10.706 patients). The results were equally positive irrespective of using educational interventions as part of the intervention or using additional counseling techniques as part of the intervention as compared to the control groups using neither of them, respectively.

#### 3.12.3. Limitations

See Section 3.13.3 for limitations of patient education in general.

### 3.13. Education in Special Patient Groups

The following recommendations are based on the most recent and topic-related scientific guidelines and by a semi-structured evaluation of scientific literature (classification: S2k; see Methods in Part 1).

#### 3.13.1. Recommendations

- Patients with coronary artery disease, congestive heart failure, peripheral arterial disease (PAD), hypertension, diabetes mellitus, and after implantation of a ventricular assist device
- Patients are recommended to receive disease-specific education during cardiac rehabilitation. (↑↑, 100%)
- Education is recommended to deliver knowledge about causes and therapy of the specific disease, to train self-management capabilities, and to promote a disease-specific healthy lifestyle as a substantial part of the risk factor management. (↑↑, 100%)
- It is suggested to include the patients’ relatives in education programs whenever possible. (↑, 100%)
- Every PAD patient is recommended to follow a special walking exercise training program including specific goals to reach the individual pain-free walking distance and to implement the vascular training program during daily life activities. (↑↑, 100%)
- It is recommended to motivate PAD patients to take part in a special vascular training group after discharge from CR. (↑↑, 100%)
- In patients who are still smoking, it is recommended to offer a cognitive behavioral group program or a behavioral oriented consultation on a face-to-face basis within a guideline-based program for smoking cessation. (↑↑, 100%)
- It may be considered to implement individual material for self-help of the patient. (↔, 100%)
- In obese patients with a BMI ≥30 kg/m^2^ or patients with a BMI of 25–25.9 kg/m^2^ exhibiting additional cardiovascular risk factors are recommended to take part in a structured and intensive lifestyle intervention. (↑↑, 100%)
- This lifestyle intervention in obese patients is recommended to include physical activity and nutritional counseling, as well as cognitive behavioral therapy. (↑↑, 100%)
- In obese patients, it is recommended to mutually define an individual target bodyweight that is about 5–10% below the initial weight and considering individual and disease-specific conditions to be reached within 6 to 12 months. (↑↑, 100%)
- The multimodal intervention towards normalization of body weight is recommended to be continued at least 6 to 12 months after CR discharge. (↑↑, 100%)

#### 3.13.2. Scientific Evidence

##### Coronary Artery Disease (CAD)

Education programs applied for secondary prevention and lifestyle intervention programs, including educational techniques, exhibited a significant reduction in total mortality (until minus 34%), cardiac mortality, and myocardial reinfarction rate. Besides, different components of a healthy lifestyle were improved [46,229,235,236,237]. A Cochrane-review about patient education being the main component of cardiac rehabilitation [238] revealed significant positive effects on total mortality (RR 0.80, 95% CI 0.60–1.05; in 13 RCTs, n = 10.075 patients), on myocardial reinfarction rate (RR 0.63, 95% CI 0.26–1.48; in 2 RCTs, n = 209 patients), on the need for revascularization (RR 0.58, 95% CI 0.19–1.71; in 3 RCTs, n = 456 patients) and on rehospitalization (RR 0.93, 95% CI 0.71–1.21; in 5 RCTs, n = 14.849 patients). Positive effects on “further cardiovascular events” and on a healthy lifestyle were seen as well (RR = 0.36, 95% CI 0.23–0.56; in 2 RCTs, n = 310 patients).

##### Congestive Heart Failure (CHF)

A meta-analysis evaluating self-management interventions [239] showed a significant reduction in the combined endpoint “time until hospitalization due to worsening heart failure” or total mortality (HR 0.80, 95% CI 0.71–0.89; in 10 RCTs, n = 3.461 patients) as well as “time until hospitalization due to worsening heart failure” alone (HR 0.80, 95% CI 0.69–0.92; in 10 RCTs, n = 3.461 patients). In addition, a significant improvement of the disease specific quality of life could be evaluated after 12 months of follow-up (SMD 0.15, 95% CI 0.00–0.30; in 11 RCTs, n = 3.356 patients).

##### Peripheral Arterial Disease (PAD)

A review including 5 RCTs and one pre/post study (n = 1.087 patients) about the effectiveness of education programs to promote physical activity and vascular exercise training revealed no clearcut effect on daily physical activity (measured by step-count or activity questionnaire) on walking distance and quality of life. The authors conclude that data are inconclusive, and more studies about education and group counseling in this specific entity with PAD are urgently needed [240].

##### Diabetes Mellitus (DM)

Meta-analyses and reviews exhibited significant positive effects of educational programs on blood glucose control, cardiovascular risk factors (body weight, blood lipids), and self-management parameters [241,242,243,244,245,246,247,248,249,250,251,252,253]. So far, there are no effects on mortality or morbidity. Another meta-analysis about self-management programs in a group setting including 47 studies (43 RCTs, 3 CCTs) revealed positive effects on blood glucose control with a reduction in the HbA1c of 0.34% (95% CI −0.51 bis −0.17; *p* < 0.0001; n = 7.055 patients) as compared to the control group (usual care) during a follow-up period until 48 months. Knowledge about diabetes disease, body weight, fasting blood glucose, triglycerides, and waist circumference were significantly improved as well [241].

##### Smoking Cessation

Patient education programs being a part of cardiac rehabilitation revealed significant positive effects on reducing of smoking habits [237,254,255]. In a meta-analysis [256] about psycho-educative interventions on smoking cessation in patients with CAD, the rate of non-smokers was 44% to 51% higher than in the control groups (point-prevalence: RR 1.44, 95% CI 1.20–1.73; in 13 RCTs; n = 3.360 patients; continuous prevalence: RR 1.51, 95% CI 1.18–1.93; in 10 RCTs; n = 2.879 patients) during a follow-up between 6 and 66 months. However, there was only a trend towards reduced total mortality (RR 0.73, 95% CI 0.46–1.15) in 10 RCTs; n = 2.593 patients.

##### Obesity

In general, education programs and cognitive-behavioral therapies are delivered within a multimodal lifestyle intervention. Meta-analyses and reviews, including RCTs and uncontrolled cohort studies, show significant positive effects of lifestyle intervention programs in patients with obesity exhibiting a reduction in the absolute body weight, the BMI or a relative (%) loss of weight [257,258,259,260]. These effects were independent of concomitant cardiovascular comorbidity. The impact on cardiovascular risk factors (blood lipids, blood pressure, HbA1c) was heterogeneous in these studies [258,259,260,261]. In obese patients exhibiting a BMI > 40 kg/m^2^ or a BMI > 35 kg/m^2^ including cardiovascular comorbidity and multimodal lifestyle-interventions, lasting at a minimum of 12 weeks, were able to reduce absolute body weight between 1.2 and 11.5 kg, BMI between 0.3 and 4.0 kg/m^2^ and relative weight between 3.1 and 6.5% during 3 to 24 months of follow-up as compared to a control group [260].

#### 3.13.3. Limitations

Education programs are usually delivered within comprehensive CR offering multiple interventions on healthy lifestyle and nonpharmacological secondary prevention. Hence, it is impossible to separate the positive effects of different interventions applied simultaneously to the patients during CR. There are also patient cohorts (e.g., with PAD, after VAD implantation) in whom data are sparse and not conclusive. Furthermore, there is limited evidence that interventions are most effective in different patient groups (e.g., age, gender, health literacy). As a consequence, more studies are warranted about the effects on clinical endpoints, and on effective patient education programs, during CR.

##### Special Patient Groups

Exercise based cardiac rehabilitation (ebCR) is usually applied to adult patients. As cardiac surgery has advanced in the last decades, more young adults with congenital heart diseases (ACHD) enter into CR, often after multiple operations during lifetime. (see Part 1 [1]) On the other hand, the number of patients above the age of 75 years is also steadily increasing [262]. Hence, frailty is becoming a more important factor during ebCR as well. In addition, there is growing evidence in the literature that gender specific differences in cardiovascular diseases also apply to CR [10,11,229,263]. As migration is increasing in our societies, other sociocultural backgrounds, and language barriers are becoming more relevant for patients in ebCR. This Chapter about “special patient goups” tries to adress these specific topics in brief and to give concrete proposals how to manage ebCR in special patient cohorts.

### 3.14. Aged and Frail Patients

The following recommendations are based on most recent and topic-related scientific guidelines and by a semi-structured evaluation of scientific literature (classification: S2k; see Methods in Part 1).

#### 3.14.1. Recommendations

- At admission to CR, it is suggested to check criteria of frailty in old and week patients. (↑, 100%)
- If frailty is diagnosed, it is suggested to implement dynamic strength training and physical exercise to reduce coordinative problems and consequently, to prevent falls. (↑, 100%)
- In old and frail patients, it is suggested to implement special nutrition advices. (↑, 100%)
- The individual and specific training during CR is suggested to be continued at home after discharge from CR. (↑, 100%)

#### 3.14.2. Scientific Evidence

Frailty is a syndrome encompassing somatic, mental, psychological and social factors. The correct diagnosis of frailty is important as it may have implications on rehospitalisation and on mortality [264] Very widely used and preferred by the authors are the criteria of frailty according to Fried et al. [265].

In aged and frail patients, significant improvements of the 6-min walk test and quality of life as measured by the MacNew Quality of Life Questionnaire, could be achieved by continuous aerobic exercise training at moderate intensity [264,266,267,268,269,270,271,272,273,274] A further increase in mobility, evaluated by the “Timed Up and Go Test”, can be a result of an additive dynamic strength training and physical exercise to improve coordinative functions [275]. Alternative forms of activity, such as Qi-Gong, are able to improve the sense of balance and reduce coordination problems significantly [276].

An individualized and specific exercise training is mandatory in this cohort, including specific components to improve mobility, coordination and strength in order to prevent falls and to reconstitute or stabilize physical capacity. In addition, malnutrition has to be looked for very carefully [277,278,279,280]. In order to maintain positive effects of CR in the long-term and to stabilize physical and mental autonomy, all activities should be continued at home [281,282].

#### 3.14.3. Limitations

The clinical relevance of specific tests to diagnose the syndrome of frailty during cardiac rehabilitation has not been validated systematically yet. In addition, there is no generally accepted tool for the diagnosis of frailty and there are no guidelines of medical societies to give advice, how frailty as diagnosed during CR has to be addressed [283]. The index of Fried et al. [265] seems to be used most widely. Other indices, however, [284] consider cognitive impairment as well.

### 3.15. Children, Adolescents and Young Patients

The following recommendations are based on most recent and topic-related scientific guidelines and by a semi-structured evaluation of scientific literature (classification: S2k; see Methods in Part 1).

#### 3.15.1. Recommendations

- In children, adolescents, and young adults, participation in CR is recommended if the disease or the intervention are likely to impair their physical or mental development or the return into social life. (↑↑, 100%)
- It is recommended to have special competence in pediatric cardiology on all levels (physicians, nurses, psychologists, physiotherapists). (↑↑, 100%)
- It is recommended to provide special groups with children, adolescents and young adults at similar age to facilitate the exchange of ideas, needs, and worries between patients. (↑↑, 100%)
- It is recommended to use a therapeutic concept that includes the specific problems of the young, especially in young patients with development delay. (↑↑, 100%)

#### 3.15.2. Scientific Evidence

Most of the patients are young adults with congenital heart disease. (ACHD, see Part 1, Section 3.15) In two studies [285,286] positive and sustainable effects of cardiac rehabilitation (CR) in children with congenital heart diseases were reported. In 19 patients with congenital heart diseases, between eight and seventeen years, an exercise based CR (ebCR) programme was performed on an outpatient basis for 12 weeks. At the end of the ebCR programme, oxygen uptake was significantly improved. The positive effect remained stable and significant until 7 months after discharge from ebCR, as compared to a control group without ebCR. These improvements in exercise capacity correlated with a better self-assessment and a better mood [285,286]. Another study [287] evaluating ebCR in children and adolescents with cardiomyopathy revealed significant improvements of quality of life, physical capacity, muscular strength and mobility, and metabolic risk factors.

In a systematic review including 31 studies, ebCR could be evaluated in 621 young patients with congenital heart diseases [288]. In most of the studies, a 12 week outpatient ebCR programme with a minimum of three ebCR sessions per week was performed. In 23 studies, a significant increase in physical activity, oxygen consumption (VO_2peak_), muscular strength. There were no negative or neutral results and in some studies, positive effects were still detectable until 6 months after discharge from ebCR [288]. Another systematic review in children and adolescents with congenital heart diseases evaluated the different components of ebCR in 16 studies of different methodological quality [289]. Aerobic continuous training and dynamic resistence training were the most frequently used exercise activities. There were no negative side effects reported. The authors state that ebCR remains underused, and scientific work should be intensified in this field to reveal the full potential of ebCR in young patients with congenital heart diseases [289].

To involve the parents, or other reference persons, in this very specific patient group is essential, if psychological or social problems exist in addition to the medical or surgical situation. Psychosocial distress, anxiety, feelings of inferiority, and missing hours in school or university are potential factors to reduce quality of life and to impair the success of ebCR [290]. In this situation, psychologists and the multidisciplinary CR-team must try to prevent retreat and social isolation of the young patients. Sometimes, psychological care of the parents or siblings might be necessary as well [291]. Physical activity and the motivation for an active and self-determined lifestyle are the core component of CR in this entity [292,293]. If residual defects maintain after surgery or intervention, specific restrictions in exercise intensity may prevail [294]. On the other hand, overprotection, a sedentary lifestyle or a protective posture should be avoided as well. In addition, educational or occupational reintegration may be further topics for the multidisciplinary team. These are the challenging tasks of CR in this specific patient cohort.

#### 3.15.3. Limitations

The scientific evidence of ebCR in young patients, adolescents or adults with congenital heart diseases (ACHD) is limited by the fact that studies are small so far, including heterogeneous medical entities and evaluating different exercise interventions [295]. Hence, recommendations are mostly based on expert opinion that is substantiated by empiric studies and shared by several professional societies [197].

### 3.16. Gender Issues

The following recommendations are based on most recent and topic-related scientific guidelines and by a semi-structured evaluation of scientific literature (classification: S2k; see Methods in Part 1).

#### 3.16.1. Recommendations

- At entry to CR, it is suggested to perform a gender-sensitive risk-evaluation, especially considering multi-morbidity, obesity, depression, and psychosocial problems. (↑, 100%)
- Secondary preventive medication is suggested to be optimised in a gender-specific way, especially considering hormone replacement therapy. (↑, 100%)
- Physical exercise and psychological groups are suggested to be offered separately for female and male patients in order to account for gender-specific preferences, thus increasing acceptance and motivation during therapy. (↑, 100%)
- During acute care in hospital, it is recommended to particularly motivate female patients to participate in cardiac rehabilitation to avoid gender specific underutilization. (↑↑, 100%)

#### 3.16.2. Scientific Evidence

Most evidence regarding sex and gender differences in cardiovascular disease exists for coronary artery disease (CAD), showing differential impact of CAD risk factors, such as smoking, diabetes, and obesity on the development and prognosis of CAD, differences in symptom presentation and underlying pathophysiology, making it more difficult to diagnose acute coronary syndrome (ACS) in women. It is also known that women have a higher age-adjusted multimorbidity at the time of diagnosis than men [296,297,298].

Though the clinical effectiveness of CR is well established [10,11,229,263], a number of international studies have shown that CR enrollment rates are significantly lower for female than for male patients [299,300,301]. Main predictors for underutilization have been a lack of strong physician recommendation, economic reasons, family responsibilities, comorbid conditions, depression, and obesity. In Germany, only about 25% of participants in cardiac rehabilitation clinics are women [302]. According to German hospital statistics 2019, the male/female ratio for patients with ACS (ICD10: I20–I25] is 2:1 in acute care and 3:0 in CR [302].

Regarding gender-specific effects of CR on cardiac risk factors, long-term morbidity and mortality, it should be noted that the relevant meta-analyses are mainly based on studies of middle-aged men, where women, as well as older men, have been underrepresented. Even if a sufficient number of women were included, data are often not analyzed separately by gender. Hence, gender-specific differences in effect sizes of CR cannot be assessed so far. This also applies to gender-specific effects of secondary preventive medication [296,303].

At entry to CR after ACS, women exhibit significantly more concomitant risk factors such as hypertension, hyperlipidemia, diabetes mellitus, and obesity than male patients [296,298]. Female patients also have a higher prevalence of depressive symptoms and anxiety, a lower physical capacity, more cardiac co-morbidity, and poorer self-rated quality of life [304,305,306].

Consequently, CR programs should be more gender-sensitive, taking into account the higher prevalence of comorbid conditions among women as well as sex-specific preferences regarding physical exercise and psychological counselling. Specific women-tailored intervention studies found that adherence to CR, quality of life, and self-rated mental health improved significantly [301,307]. Another trial showed that female patients felt more comfortable when they exercised in women-only groups, and perceived the environment less competitive than in mixed-gender groups [308]. And women particularly preferred supervised exercise training over home-based CR programs. In a controlled clinical study in Germany with in-hospital CR patients it was found that exercise training, psychological, and nutritional counselling of female patients, in women-only groups, resulted in a significant improvement in exercise testing (from 74.1 watt at entry to 83.2 watt at discharge; *p* < 0.05) whereas the respective increase among women in the control group of mixed-gender (72.9 watt to 76.9 watt) was statistically insignificant [304,306]. Twelve months after discharge from in-hospital CR, 82% of patients of the initial women-only group still exercised physically more than 60 min per week compared to 73% of women of the mixed-gender group (*p* < 0.05) [306,309]. According to a systematic review, subjective assessment and awareness of individual coronary risk factors also seems to vary by sex [310]. The review found that male CAD patients were more convinced that their own behavior had caused their disease than women. This fact should be given more attention during CR. Significant changes in health-related behavior can better be achieved if gender-tailored education and counselling is applied. In general, women seem to benefit especially from psychosocial and emotional support, whereas men prefer a more competitive CR setting, emphasizing physical exercise.

#### 3.16.3. Limitations

Current research suggests that gender-tailored CR enhances adherence, satisfaction with care, effectiveness of exercise training, changes in health behavior, and self-rated mental health. Further prospective studies and clinical trials with representative patient cohorts and different cardiovascular diseases are necessary to assess, more accurately, the impact of gender-tailored CR on specific clinical endpoints.

### 3.17. Patients with Migration Background

The following recommendations are based on the most recent and topic-related scientific guidelines and by a semi-structured evaluation of scientific literature (classification: S2k; see Methods in Part 1).

#### 3.17.1. Recommendations

- It is suggested that rehabilitation facilities provide information for patients in commonly spoken foreign languages and intercultural training for staff to gain intercultural competence. (↑, 100%)
- It is suggested that multidisciplinary teams work together with professional translators and professional mediators to moderate intercultural differences. (↑, 100%)
- In the long-term, it is suggested to establish diversity-sensitive structures in order to recognise and to interact with differing expectations and needs of patients, independently of a potential migration background. (↑, 91%)

#### 3.17.2. Scientific Evidence

Persons who immigrated themselves, or whose parents immigrated, are said to have a migration background, irrespective of their citizenship. In Germany, almost 25% of the population have a migration background [311]. On average, persons with a migration background have a lower socioeconomic status and more often work in occupations with a higher physical and psychological burden [312,313]. Data show that patients with a migration background have a lower probability to participate in cardiac rehabilitation (CR), even after adjusting for age as well as demographic and socioeconomic confounders [314]. Aside from a limited health literacy and poor German language proficiency [315], these differences may result from services not being sufficiently sensitive to the diverse needs and expectations of migrants [316]. Similar findings were reported from other European countries [317]. Unlike migrants who reside in Germany for many years, newly arrived refugees and asylum seekers, without a residence status, encounter additional barriers to accessing health care because, initially, they are only entitled to a limited set of services [318].

In Germany, studies about CR in patients with migration background are sparse, and their results are conflicting. An evaluation of routine data of a pension fund between 2000 and 2006 showed no difference between Turkish and non-Turkish patients in terms of their treatment outcome after CR [319]. Another analysis of pension fund data, between 2002 and 2009, revealed that Turkish participants in CR were less satisfied with the course of their CR, and they reported a lower self-reported health status after CR. In addition, Turkish participants in CR had a 60% higher risk to be unemployed after CR as compared to German participants [316].

As a consequence, continuous education and training is necessary to raise awareness among staff with respect to intercultural differences and to strengthen the intercultural competence of the interdisciplinary team towards patients with migration background. The opening of CR to diversity-sensitive thinking and acting will help to reduce not only challenges related to migration background but also to other markers of diversity such as gender, skin colour, or religious affiliation [320].

#### 3.17.3. Limitations

Prospective studies investigating the referral or utilization of cardiac rehabilitation as a function of migration background or citizenship have not been performed so far. Data from health insurances or pension funds, where available, are descriptive only. Hence, further studies are needed to evaluate the clinical relevance of migration background on referral to CR and on the prognosis of cardiovascular diseases. As migration, social systems, health systems, and societies in general do differ between countries, these issues have to be studied in every country separately, and results may not be generalizable.

### 3.18. Telemedicine in Cardiac Rehabilitation and Home-Based Rehabilitation

The following recommendations are based on the most recent and topic-related scientific guidelines and by a semi-structured evaluation of scientific literature (classification: S2k; see Methods in Part 1).

#### 3.18.1. Recommendations

- Cardiac rehabilitation of phase II is recommended to be preferably offered under face-to-face supervision and responsibility of a multidisciplinary rehabilitation team (centre based CR). (↑↑, 100%)
- It is suggested to establish tele-rehabilitation facilities for low risk patients with sufficient time interval since a non-complicated index-event to support cardiac rehabilitation participation in general. (↑, 100%)
- It is suggested to establish home-based rehabilitation to intensify long-term secondary prevention of patients with cardiovascular disease. (↑, 100%)

#### 3.18.2. Scientific Evidence

In seven systematic reviews with meta-analyses [321,322,323,324,325,326,327] and one Cochrane review [328], randomised controlled trials as well as cohort studies were evaluated. Concerning clinical endpoints of mortality, cardiovascular event rate, physical activity, quality of life, smoking cessation, reduction in blood pressure, and LDL-cholesterol, there was no significant difference between tele-rehabilitation and conventional cardiac rehabilitation (CR) during a follow-up time between 3 and 12 months [321,322,323,324,325,326,327,328].

The largest meta-analysis [326] included 60 randomised controlled trials (RCT’s) with a total of 19.411 patients with coronary artery disease (CAD). As compared to usual care, total mortality (RR 0.76; 95% CI 0.64–0.90; *p* = 0.002), and cardiovascular mortality (RR 0.65; 95% CI 0.64–0.90; *p* = 0.002) were significantly reduced by center-based CR (hospital-based or outpatient clinic) only. The endpoints myocardial infarction, hospital admission, CABG-surgery or coronary intervention were similar between centre-based-, home-based-, or tele-CR as compared to usual care [326].

In the Cochrane review, 23 RCT’s including 2.890 patients, predominantly with chronic CAD, were analysed [328]. In five studies, patients after acute coronary syndrome (ACS) and in four studies, patients after CABG-surgery or with congestive heart failure (CHF) were included [328]. Only home-based- and centre-based-CR were compared, and there was no usual care group. Mortality, physical activity, and quality of life (QoL) did not differ between groups. There was no separate analysis for patients with chronic or acute CAD, CABG, or CHF [328].

Three RCT’s evaluated tele-CR thereafter. In 850 patients with CHF, a 9-week comprehensive tele-CR programme was safe and QoL as well as exercise capacity were significantly improved at week 9. Hospitalization and mortality, however, did not differ from control until 26 months (TELEREH-HF) [329]. In 120 patients with cardiovascular disease and after a conventional 4 week outpatient-CR, an additional 6 months home-based exercise training was safe. System use decreased over time, and at month 6, physical activity time was significantly higher, whereas oxygen consumption (VO_2peak_), QoL, and healthy diet was not different from control (PATHway-I) [330]. Out of 4.236 Patients screened, 179 patients (4.2%) were randomized to home-based mobile CR or to control group [331]. After 12 months, primary endpoint could be evaluated in 151 patients. Oxygen consumption (VO_2peak_) improved in the home-based group (+1.2; 95% CI 0.4 to 2.01 mL/min/kg) but not in the control group (+0.1; 95% CI −0.5 to 0.7 mL/min/kg). Adverse events and quality of life did not differ between groups. (EU-CaRE).

Telemedical CR studies in CAD patients only included low risk patients after uncomplicated infarction, at the earliest 4 weeks after the index event or much later, at 3 months. Patients were excluded if they did not reach a certain work load during ergometry prior to start tele-CR, if LVEF was below 40%, patients with CHF, atrial fibrillation, diabetes mellitus, or after cardiac surgery and with an age above 75 years [321,322,323,324,325,326,327,328]. In some studies, centre-based CR was done first and tele-CR was started, when the patients were stable [332,333]. The few studies in CHF, predominantely patients in functional class NYHA II, were included, and patients with ICD- or CRT-systems were excluded [334]. There is one study that started tele-CR in 47 patients on day 4 after uncomplicated CABG-surgery (EuroScore 0–10) using bicycle exercise training at home [335]. In 7 patients (15%) home-based-CR had to be stopped early due to atrial fibrillation (n = 4), pleural effusion (n = 2), and cerebral ischemia (n = 1) [335]. The problem of maintaining sufficient security for patients shortly after CABG-surgery or ACS is demonstrated by the fact that studies did include patients only, if they were living within a certain distance from the next chest pain unit, and if there was a second person being permanently in the home-based training room to give first aid in case of an cardiac emergency during tele-CR [334]. Adherence to CR or cost effectiveness does not seem to be significantly different between tele- or home-based-CR as compared to centre-based- or outpatient-CR [328,332,333,336,337].

ACC/AHA has postulated goals for CR in general that have to be evaluated concerning reduction in clinical endpoints, adherence to CR-activities, and fulfillment of quality measures. In addition, participation rate of patients during tele-CR must be documented, and CR should be started no later than 21 days after the index event [338]. EAPC, ESC, and AACVPR stated that tele-CR is a valuable tool to implement secondary prevention in cardiovascular patients more widely and effectively [338,339,340,341,342]. In rural areas, however, availibility, stability, and acceptance of modern telemedical techniques are in need of improvement [343]. Hence, the CR-setting, centre-based-, outpatient-, tele-, or home-based- should be chosen in dependance of the severity of the disease, the local availiblity and the national guidelines [344]. During long-term secondary prevention, physical capacity, as measured with VO_2peak_ and with the Physical Activity Score for the Elderly (PASE), was significantly increased by home-based-exercise intervention supported by telephone calls as compared to a control group until 6 years after CABG-surgery [345].

#### 3.18.3. Limitations

Most of the tele-CR studies included only exercise training, and comprehensive or multidisciplinary CR was not performed. In addition, studies did not give exact information about comprehensive CR activities or frequency, duration, and intensity of exercise training [326]. In some tele-CR studies, exercise training was defined as “activities that the patient could perform alone at home, such as walking around” and occasionally, patients were supported by trained personel in the tele-CR group [321,322,323,324,325,326,327,328]. This methodological heterogeneity [321,322,323,324,325,326,327,328] precludes generalization of study results [346,347,348,349]. In addition, predominantly low-risk patients have been evaluated in tele-CR studies so far, and there are no standards for selecting patients that are suitable for tele- or home-based-CR. Hence, it is questionable whether clinical results derived from studies in coventional CR may be transferred to tele-CR [350,351]. At the moment, there are several tele-CR studies on the way addressing these open issues. (CR-GPS, eEduHeart I, SmartCare-CAD) Tele-CR studies on psychosocial effects or the impact on return-to-work are very sparse [352], and technical problems might cause a very low adherence to CR activities in the tele-CR group [353].

## 4. Summary and Critical Conclusions

The guidelines on cardiac rehabilitation (CR) in German speaking countries (LLKardReha-DACH) aim to summarize the actual scientific evidence with respect to CR-indications (Part 1; (1) and CR-delivery (Part 2). Physical activity and exercise training are the cornerstones of CR with evidence for reduction in mortality in patients with coronary artery disease and with evidence for improvements of morbidity, exercise capacity, and quality of life in patients with congestive heart failure, valve surgery, or intervention, after VAD-implantation or heart transplantation, in adults with congenital heart disease and peripheral arterial disease. Prognostic impact of exercise training has not yet been detected in these entities. Based on heterogeneous studies, psychological interventions did not show a significant incremental positive effect when added to “exercise-based rehabilitation” in patients with coronary artery disease and congestive heart failure. Regarding specific interventions for “distress management” and “lifestyle change” there was a positive trend towards reduction in depressive symptoms. Clinical endpoints, such as morbidity, mortality, and quality of life could not be evaluated due to small sample size and heterogeneity of these studies. Hence, distinct psychological interventions during CR should be adjusted to the needs of the individual patient. Patient education is able to increase knowledge and motivation, as well as behavior changes, regarding physical activity, dietary habits, and smoking cessation. As education programs offer multiple interventions on healthy lifestyle and nonpharmacological secondary prevention, there is limited evidence which interventions are most effective in different patient groups (e.g., age, gender, health literacy). In addition, there are patient cohorts (e.g., with PAD, after VAD implantation) in whom data are sparse and not conclusive. Hence, further studies are warranted about the effects of education during CR on clinical endpoints. The evidence for distinct CR programs in special patient groups is less clear as well. Diversity-sensitive structures, however, should be established in CR to interact with the needs of special patient groups and gender issues. Studies on telemedical or home-based CR predominantly included low-risk patients, mostly weeks or months after the index event. Hence, it is questionable whether clinical results derived from studies in conventional centre-based or outpatient CR may be transferred to Tele-CR. As these new forms of CR-delivery are valuable tools to facilitate referral to CR or to implement CR in remote areas, tele-CR should be further investigated.

## Figures and Tables

**Table 1:** Grades of recommendation (for details see Part 1; [1]).

Strong recommendation	“is recommended…” ↑↑ “is not recommended…” ↓↓
Medium recommendation	“is suggested…” ↑ “is not suggested….” ↓
neutral	“may be considered” ↔

**Table 5:** Meta-analyses until 2018 on psychological intervention in cardiovascular disease.

Study-Design	Number of Studies	Population	Results: (+) = Effective; (0) = Effectivity Not Proven (SMD, MD, OR, log-OR, d, ARR, *r* (95% CI))	Annotations
[215]				
RCTs	12	N = 2.202 CHD, ACS/MI	**Depression SMD −0.35 (−0.52; −0.17) (+)** **(depressive symptoms reduced)** **Anxiety SMD −0.34 (−0.65; −0.03) (+)** **(anxiety reduced)** **QoL mental MD 3.62 (0.2; 7.02) (+)** **(mental quality of life improved)** QoL somatic MD 2.59 (−0.41; 5.60) (0) CV events OR 0.80 (0.3; 1.93) (0) treatment satisfaction SMD 0.11 (−0.29; 0.51) (0)	Studies 2003–2014. Patients with higher depressive symptoms and anxiety. CBT only +/− antidepressive medication. Low study-quality, control and intervention group inhomogeneous
[216]				
RCTs	35	N = 10.703 CAD, AMI, PCI, CABG,	**Depression SMD −0.27 (−0.39; −0.15) (+)** **(depressive symptoms reduced)** **Anxiety SMD −0.24 (−0.38;−0.09) (+)** **(anxiety reduced)** **Disstress SMD −0.56 (−0.88; −0.24) (+)** **(disstress reduced)** **Cardiac mortality RR 0.79 (0.63; 0.98) (+)** **(Cardiac mortality reduced)** Total mortality RR 0.90 (0.77; 1.05) (0) PCI/CABG RR 0.94 (0.81; 1.11) (0) Non-fatal MI RR 0.82 (0.64; 1.05) (0)	Studies 1974–2016. Low study-quality, control and intervention group inhomogeneous
[217] (results on “mental health treatments“ only)				
RCTs	18	N = 9.819 CHD, CHF	**Depression *d* 0.297 (0.16; 0.43) (+)** **(depressive symptoms reduced)** Total mortality ARR −0.001 (−0.016; 0.15) (0) **Cardiovascular mortality and events ARR 0.029 (0.007; 0.51) (+)** **(Cardiovascular mortality and events reduced)**	Studies 1996–2011 Psychological intervention without antidepressive medication. Low study-quality, control and intervention group inhomogenous
[218]				
RCTs	51	N = n. a. CHD	**Depression SMD −0.23 (−0.35; −0.11) (+)** **(depressive symptoms reduced)** **Anxiety SMD −0.15 (−0.29; −0.04) (+)** **(anxiety reduced)** Total mortality log-OR −0.14 (−0.47; 0.15) (0) Cardiac Mortality log-OR −0.16 (−0.44; 0.07) (0) **Non-fatal MI log-OR −0.35 (−0.65; −0.10) (+)** **(non-fatal MI reduced)**	Studies 1974–2006 Control and intervention group inhomogeneous
[214]				
RCTs	43	N = 12.023 CHD, MI, PCI, CABG	Depression (Int vs. Co) *r* −0.30 vs. −0.21 (0) **Depression men (Int vs. Co) *r* −0.28 vs. −0.17 (+)** **(Depression in men reduced)** Depression women (Int vs. Co) *r* −0.28 vs. −0.23 (0) Anxiety (Int vs. Co) *r* −0.17 vs. −0.11 (0) Anxiety in men (Int vs. Co) *r* −0.11 vs. −0.03 (0) Anxiety in women (Int vs. Co) *r* −0.18 vs. −0.25 (0) Disstress (Int vs. Co) *r* −0.36 vs. −0.20 (0) Disstress in men (Int vs. Co) *r* −0.76 vs. −0.53 (0) **Disstress women (Int vs. Co) *r* −0.27 vs. −0.10 (+)** **(Disstress in women reduced)** **Social support (Int vs. Co) *r* −0.28 vs. −0.2 (+)** **(social support improved)** **Social support men (Int vs. Co) r −0.29 vs. −0.14(+)** **(social suppoprt in men improved)** **Social support women (Int vs. Co) *r* −0.44 vs. −0.14 (+)** **(social support in women improved)** **QoL (Int vs. Co) *r* −0.21 vs. −0.13 (+)** **(quality of life improved)** SBD (int vs. Co) r −0.03 vs. 0.08 (0) DBD (Int vs. Co) r −0.09 vs. 0.03 (0) **Total mortality (<2 years) OR 0.72 (0.56; 0.94) (+)** **(total mortality improved until 2 years)** Total mortality (>2 years) OR 0.89 (0.80; 1.01) (0) **Total mortality men OR 0.73 (0.57; 1.00) (+)** **(total mortality in men reduced)** Total mortality in women OR 1.01 (0.87; 1.72) (0)	Studies 1975–2005 Control and intervention group inhomogeneous

Abbreviations: RCT: Randomized Controlled Trial; CBT: Cognitive Behavioral Therapy; CHD: (stable) Coronary Heart Disease; MI/ACS: Myocardial infarction/Acute Coronary Syndrome; PCI: Percutaneous Coronary Intervention; CABG: Coronary Artery Bypass Graft Surgery; CHF: Congestive Heart Failure; Int: Intervention-group; Co: Control-group; SMD: Standardized Mean Difference; MD: Mean Difference; OR: Odds Ratio; log-OR: logarithmic Odds Ratio; ARR: Absolute Risk Reduction; *r*: Correlation coefficient for change (converted from Cohen’s d); d: Cohen’s d; (significat results in bold).

**Table 6:** Meta-analysis on psychological interventions as add-on to exercise based cardiac rehabilitation.

Endpoints	Intervention	Number of Patients n (I; C)	Heterogeneity	SMD	95% CI	Annotations
depressive symptoms	LC	165; 159	*I*^2^ = 60%	−0.19	−2.89; 2.51	2 studies f/u 12 m
	DM	266; 237	*I*^2^ = 0%	−0.19	−0.47; 0.10	4 studies f/u 6–12 m
	LC + DM	273; 226	*I*^2^ = 58%	0.03	−0.56; 0.61	3 studies f/u 6–12 m
	all interventions	694; 622	*I*^2^ = 44%	−0.13	−0.30; 0.05	9 studies f/u 6–12 m
anxiety	LC	52; 62	n.a.	0.04	−0.33; 0.40	1 study f/u 12 m
	DM	256; 232	*I*^2^ = 0%	−0.11	−0.29; 0.06	1 study f/u 12 m
	LC + DM	57; 59	n.a.	0.43	0.07; 0.80	1 study f/u 6 m
	all interventions	365; 355	*I*^2^ = 37%	0.01	−0.24; 0.27	6 study f/u 6–12 m

Endpoints depressive symptoms and anxiety are displayed separately for each kind of intervention. All statistics according to random effects model. Abbreviations: LC: Lifestyle change intervention; DM: Distress management; I: Intervention; C: control-intervention; SMD: standardized mean difference; CI: confidence interval; f/u: follow up; m = months, n.a.: not available, I²: measure of heterogeneity according to Higgins und Thompson 2002 [223].

## Data Availability

Not applicable.

## Supplementary Materials

The following are available online at https://www.mdpi.com/article/10.3390/jcm10143071/s1. Data S1: Classification of scientific evidence according to SIGN; Table S1. Absolute and relative contraindications to physical activity and exercise training within cardiac rehabilitation (adapted from [2,6,37,354]); Table S2. Content of LL-KardReha-DACH and levels of evidence generation in its original form in German language.

## Abbreviations

ACHD	adults with congenital heart disease
ACS	acute coronary syndrome
AWMF	Association of the Scientific Medical Societies in Germany
BMI	body mass index
BORG/RPE	Borg Rating of Perceived Exertion Scale
CABG	coronary artery bypass grafting
CAD	coronary artery disease
CCS	chronic coronary syndrome
CCT	controlled cohort trial
CHF	congestive heart failure
CR	cardiac rehabilitation
CRT	cardiac resynchronization therapy
CV	cardiovascular
CVD	cardiovascular disease
cTGA	corrected transposition of great arteries
DM	diabetes mellitus
ebCR	exercise based cardiac rehabilitation
ECG	electrocardiogram
EF	ejection fraction
ET	exercise training
HADS	Hospital Anxiety and Depression Scale
HFmrEF	heart failure with mid-range left ventricular ejection fraction
HFpEF	heart failure with preserved left ventricular ejection fraction
HFrEF	heart failure with reduced left ventricular ejection fraction
HIIT	high intensity interval training
HR	heart rate
HRpeak	heart rate at peak exercise
HRR	heart rate reserve
HTX	heart transplantation
ICD	implanted cardioverter defibrillator
IMT	inspiratoric muscle training
IT	interval training
LDL	low density lipoprotein
LV-EF	left ventricular ejection fraction
MCT	moderate continuous training
MET	metabolic equivalent
NSTEMI	non ST-elevation myocardial infarction
NYHA	New York Heart Association
QoL	quality of life
PA	physical activity
PAD	peripheral artery disease
PCI	percutaneous coronary intervention
PE	patient education
PI	psychological intervention
PTA	peripheral transluminal angioplasty
RCT	randomized controlled trial
RM	one repition maximum
STEMI	ST-elevation myocardial infarction
TEA	thrombendatherectomy
Tele	telemedical
TIA	transitoric ischemic attack
VAD	ventricular assist device
VO_2peak_	oxygen consumption at peak exercise
VT1	ventilatory threshold 1
VT2	respiratory compensation point
WIQ	walking impairment questionnaire score
WCD	Wearable Cardioverter-Defibrillator
