# Supplementary material for: Cardiac Rehabilitation in German Speaking Countries of Europe—Evidence-Based Guidelines from Germany, Austria and Switzerland LLKardReha-DACH—Part 2
Source: J Clin Med. 2021 Jul 12;10(14):3071. doi: 10.3390/jcm10143071 (PMC8306118; doi:10.3390/jcm10143071)
Supplement: Supplementary file 1 [file jcm-10-03071-s001.zip › jcm-1236095-supplementary.pdf]

Supplementary Materials

Data S1:

Classification of scientific evidence according to SIGN
Ref.: Scottish Intercollegiate Guidelines Network, SIGN 50, "A guideline developer's handbook". https://www.sign.ac.uk/pdf/sign50.pdf

Levels of evidence

1 ++ High quality meta-analyses, systematic reviews of RCTs or RCTs with a very low risk of bias

1 + Well conducted meta-analyses, systematic reviews or RCTs with a low risk of bias

1 - Meta-analyses, systematic reviews or RCTs with a high risk of bias

2 ++ High quality systematic reviews of case control or cohort studies. High quality case control or cohort studies with a very low risk of confounding or bias and a high probability that the relationship is causal

2 + Well conducted case control or cohort studies with a low risk of confounding or bias and a moderate probability that the relationship is causal

2 - Case control or cohort studies with a high risk of confounding or bias and a significant risk that the relationship is not causal

3 Non-analytic studies, e.g. case reports, case series

4 Expert opinion

Table S1. Absolute and relative contraindications to physical activity and exercise training within cardiac rehabilitation (adapted from (2, 6, 37, 350)).

**Note: the list of contraindications given below does not claim to be exhaustive and does not relieve the responsible physician of his or her own responsibility for clinical examination and decision-making**	
Absolute contraindications	• acute coronary syndrome (ACS) < 48 hours<br>• known untreated and clinically relevant main stem stenosis<br>• uncontrolled cardiac arrhythmias at rest or during exercise<br>• severe aortic valve stenosis or other severe valvular heart disease<br>• decompensated heart failure<br>• acute pulmonary embolism or pulmonary infarction<br>• acute aortic dissection<br>• acute non-cardiac conditions that limit exercise capacity or are worsened by physical exertion (e.g., infection, sepsis, hyperthyroidism/ hypothyroidism)<br>• acute deep vein thrombosis<br>• acute myocarditis or pericarditis<br>• active endocarditis<br>• recent stroke or TIA<br>• physical disability that prevents safe and appropriately dosed exercise training<br>• other clinical situations that worsen under physical exertion
Relative contraindications	• significant anemia<br>• severe, disease-relevant electolytic derailments<br>• therapeutically uncontrolled tachy- or bradycardia/arrhythmias, e.g.<br>- untreated sick sinus syndrome<br>- untreated, higher-grade AV blockages<br>- atrial fibrillation with uncontrolled ventricular rate<br>• HOCM with maximum resting LVOT gradient > 25 mmHg<br>• known aortic dissection (Stanford B type, stable)<br>• severe arterial hypertension at rest (systolic blood pressure ≥ 180 mm Hg and diastolic blood pressure ≥ 110 mmHg)<br>• blood pressure drop of systolic ≥ 20 mmHg during exercise<br>• uncontrolled diabetes mellitus<br>• cognitive impairment that prevents/significantly limits cooperation during exercise testing and

	training<br>• relevant pericardial effusion

Table S2. Content of LL-KardReha-DACH and levels of evidence generation in its original form in German language

1. General information	- Initiation of the guideline and responsibilities<br>- Development, methods and internal reviewing processes
2. Introduction	- Aims of cardiac rehabilitation<br>- Definition of cardiac rehabilitation<br>- Legal regulations and CR-structures in Germany, Austria and Switzerland
3. General objectives of CR	- Goals and conflicting goals (NER)<br>- Cardiovascular prevention (NER)<br>- Psychosocial goals and tasks (NER)<br>- Social and vocational reintegration of patients (NER)
4. Indications for CR initiation and participation	- **after acute coronary syndrome (S3)**<br>- **after coronary bypass surgery (S3)**<br>- with chronic coronary syndrome (S2k)<br>- with high CV-risk (S2k)<br>- **with chronic heart failure (S3)**<br>- after surgical or interventional valve repair (S2k)<br>- after ICD/CRT implantation (S2k)<br>- with ventricular assist device (S2k)<br>- after heart transplantation (S2k)<br>- after surgical/interventional repair of the aorta (S2k)<br>- with chronic peripheral arterial disease (S2k)<br>- after pulmonary embolism (S2k)<br>- with chronic pulmonary hypertension (S2k)<br>- after myocarditis (S2k)<br>- adults with congenital heart disease (S2k)
5. CR-specific contents and interventions	- Medical supervision and nursing (S2k)<br>- Physical exercise modalities as adapted to the individually underlying cardiovascular diseases (S2k)<br>- **Psychological interventions (S3)**<br>- Smoking cessation (S2k)<br>- Nutrition (S2k)<br>- Ergotherapy (S2k)<br>- Social interventions (S2k)<br>- Information, education and training for lifestyle changes (S2k)
6. CV risk diseases and co-morbidities	- Hypertension (NER)<br>- Hyperlipidemia (NER)<br>- Diabetes and metabolic syndrome (NER)<br>- Adipositas (NER)<br>- Chronic renal disease (NER)<br>- Chronic obstructive lung disease (NER)<br>- Orthopaedic disorders (NER)<br>- Rheumatic diseases (NER)<br>- Psychiatric and neurological diseases (NER)
7. Special groups of patients in CR	- Old and frail patients (S2k)<br>- Young patients (S2k)<br>- Gender specialities (S2k)<br>- Migrants (S2k)
8. Medical aftercare, prevention programs	- Phase 3 programs (S2k)<br>- Integrated ambulatory health care and disease management programs (S2k)
9. Special concepts	- Work related prevention programs (S2k)<br>- Work related rehabilitation programs (S2k)<br>- Tele-rehabilitation and home-based rehabilitation (S2k)
10. Quality measurement	- Evidence – based quality assurance in CR (S2k)
**S3** = highest level of scientific evidence based on newly performed structured reviews and meta-analyses (text in bold); **S2k** = medium level of scientific evidence based on semi-structured literature searches and evaluation without performing meta-analyses; **NER** = Narrative evidence reporting based on the most actual topic-related international guidelines	
